# Halogen-Bond-Assisted Photoluminescence Modulation in Carbazole-Based Emitter

**DOI:** 10.1038/s41598-018-32830-3

**Published:** 2018-09-26

**Authors:** Jagadish K. Salunke, Nikita A. Durandin, Tero-Petri Ruoko, Nuno R. Candeias, Paola Vivo, Elina Vuorimaa-Laukkanen, Timo Laaksonen, Arri Priimagi

**Affiliations:** Laboratory of Chemistry and Bioengineering, Tampere University of Technology, P.O. Box 541, FI-33101 Tampere, Finland

## Abstract

Halogen bonding between a carbazole-based, pyridine-substituted organic semiconductor and a common halogen-bond donor (pentafluoroiodobenzene) yields efficient halogen-bond-driven fluorescence modulation in solution. Steady-state, time-resolved emission and absorption spectroscopy as well as density functional theory studies demonstrate that the fluorescence modulation arises from halogen-bond-induced intramolecular charge transfer. Fluorescence modulation offers a range of possibilities both in solution and in the solid state, for instance providing a potential pathway for the design of tunable luminescent materials for light-emitting devices.

## Introduction

Organic luminescent materials are central to a wealth of functional devices, ranging from organic light-emitting diodes (OLEDs) and sensor elements to photonic components and imaging systems^1–4^. In addition to achieving efficient luminescence, obtaining on-demand control over the emission properties, or rendering the emission responsive to changes in the environment (or the presence of analytes), is of great pertinence. The optical properties of solid-state molecular materials depend profoundly on the intermolecular interactions and molecular packing^5,6^, which can be manipulated through thermal, mechanical, or light stimuli^7–10^. In solution, fluorescent chemosensors keep attracting considerable attention, particularly for biotechnological applications^11–16^. Radiative decay engineering based on supramolecular concepts, in turn, paves the way for biological imaging^17^ and even for improving the performance of dye lasers^18,19^. In all the aforementioned cases, it is important to comprehend the supramolecular interactions between the molecular components and to understand their role in modulating the luminescence properties of the emitters.

In recent years, halogen bonding (XB) has emerged as a prominent noncovalent interaction for the design of supramolecular photofunctional materials^20,21^. It is defined as an attractive interaction between an electrophilic region associated with a halogen atom in a molecule, and a nucleophilic site^22^. Halogen bonding possesses several attractive features for supramolecular crystal engineering, such as high directionality and tunable interaction strength^23,24^. Furthermore, unlike hydrogen bonding, XB is hydrophobic in nature, thereby showing great potential not only in anion recognition and sensing^25–27^, but also in anion transport^28,29^.

The most important characteristic of XB from the perspective of light-emitting materials relates to the large size of the bond-donating atoms (bromine, iodine), which has led to the development of halogen-bonded cocrystals exhibiting efficient room-temperature phosphorescence emission^30–32^. This, combined with the fact that the emission can be effectively tuned via XB-based crystal engineering^33^, points out the vast potential of halogen bonding in luminescence control. However, examples of halogen-bond-assisted emission modulation in solution for conjugated organic molecules remain scarce^34–36^, and are to the best of our knowledge non-existent in neutral systems. Herein, we show that halogen-bond-induced intramolecular charge (ICT) in carbazole derivatives efficiently modulates their fluorescence emission in solution. We believe this finding to be important for rationally designing solid-state halogen-bonded emissive materials.

## Results and Discussion

**Figure 1:**
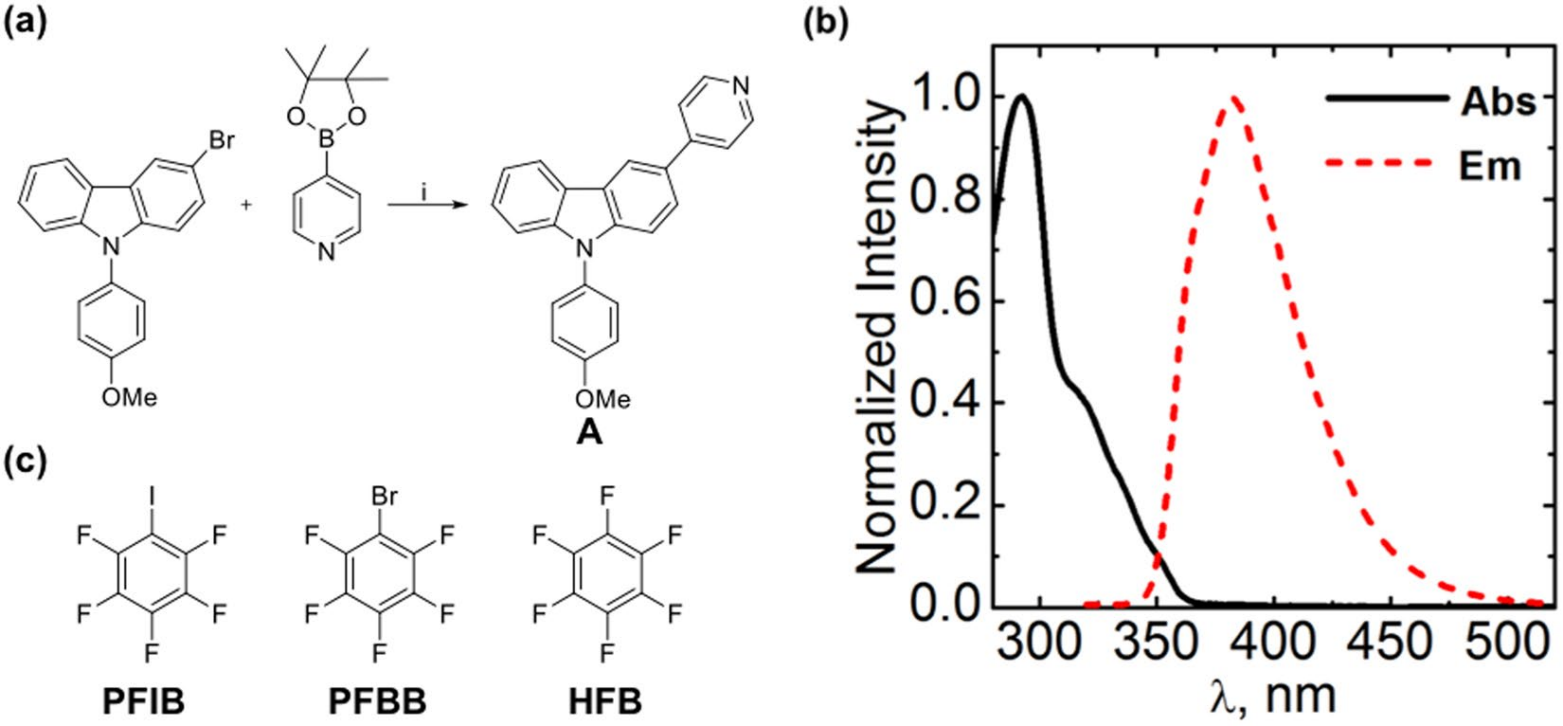
**(a**) Synthesis of **A** using Suzuki-coupling reaction. Reagents: **i**. 4-Pyridineboronic acid pinacol ester, Pd(PPh_3_)_4_, THF/Toluene (2:3), 2 M K_2_CO_3_, reflux, 24 hr, 70% yield. **(b)** Normalized absorption and emission spectra of **A** in DCM (10^−5^ M concentration). **(c)** The XB donors pentafluoroiodobenzene (PFIB) and pentafluorobromobenzene (PFBB), and the nonbonding reference compound hexafluorobenzene (HFB).

The emitter used, 9-(4-methoxyphenyl)-3-(pyridin-4-yl)-9H-carbazole (**A**, Fig. 1a), contains an electron-rich carbazole core, chosen thanks to its optical, thermal and electrochemical properties and easily tunable molecular structure^37–40^. The central carbazole moiety is also expected to increase the electron density of the XB-accepting pyridine unit, thereby enhancing its Lewis-basic character. **A** was obtained by using a simple one-step Suzuki coupling reaction of a previously reported bromo-substituted carbazole^39^ with a commercially available pyridine boronic-acid pinacol ester in 70% yield (Fig. 1a; further details given in the SI). It is highly soluble in common organic solvents, such as toluene, dichloromethane (DCM), acetonitrile (ACN), and tetrahydrofuran (THF).

In DCM, **A** exhibits two absorption bands at around 293 nm (39,000 M^−1^ cm^−1^) and 318 nm (16,000 M^−1^ cm^−1^), as shown in Figs 1b and S3. The 293 nm band corresponds to the π → π* transition of the carbazole core, whereas the 318 nm shoulder is attributed to the n → π* transition^41,42^. The fluorescence spectrum of **A** upon excitation at 320 nm shows a broad emission band with a maximum at 383 nm, attributed to the local excited state emission of carbazole. The fluorescence quantum yield (Φ_f_) of **A** was determined in four solvents with different polarity indices (P), yielding 0.34 in toluene (P = 2.4), 0.30 in DCM (P = 3.1), 0.35 in THF (P = 4.0), and 0.21 in ACN (P = 5.8) using unsubstituted carbazole as a reference. Minor deviation of Φ_f_ values for toluene, THF, and DCM are attributed to small changes in solvation energy of **A** in the solvents used. The drastic decrease of Φ_f_ in ACN can be ascribed to the strong solvation effect and higher probability of the ICT process in high-polarity solvent, leading to a fraction of non-emissive ICT state. This phenomenon has been already reported in earlier studies for the similar type of molecules^43,44^.

Halogen bonding in solution has been widely utilized in anion recognition and sensing^25–28,34,45^, and the thermodynamics of neutral halogen-bonded systems is widely elaborated^46–48^. The reported binding constants, measured typically via NMR spectroscopy, range between 1–100 M^−1^ for small molecules^47,49^ and up to 10^8^ M^−1^ for supramolecular capsules^50^. Particularly, strong binding between organic semiconducting molecules and XB donors can affect their solid-state optoelectronic properties drastically^51,52^. Our design assumption is that the Lewis-basic character of the pyridine moiety is enhanced due to the carbazole core of **A**, which will in turn strengthen the interaction with XB donors to the extent that the optical properties of neutral organic semiconducting molecules can be modulated even in solution. Indeed, based on density functional theory (DFT) calculations, the electron donating character of the carbazole moiety, when placed in the 4-pyridyl position, results in 9% increase in the halogen-bond strength when comparing the interaction of pyridine and **A** with pentafluoroiodobenzene (PFIB; see SI for further computational details). This provides a good starting point for studying the complexes between **A** and the XB donors shown in Fig. 1c^53–56^.

**Figure 2:**
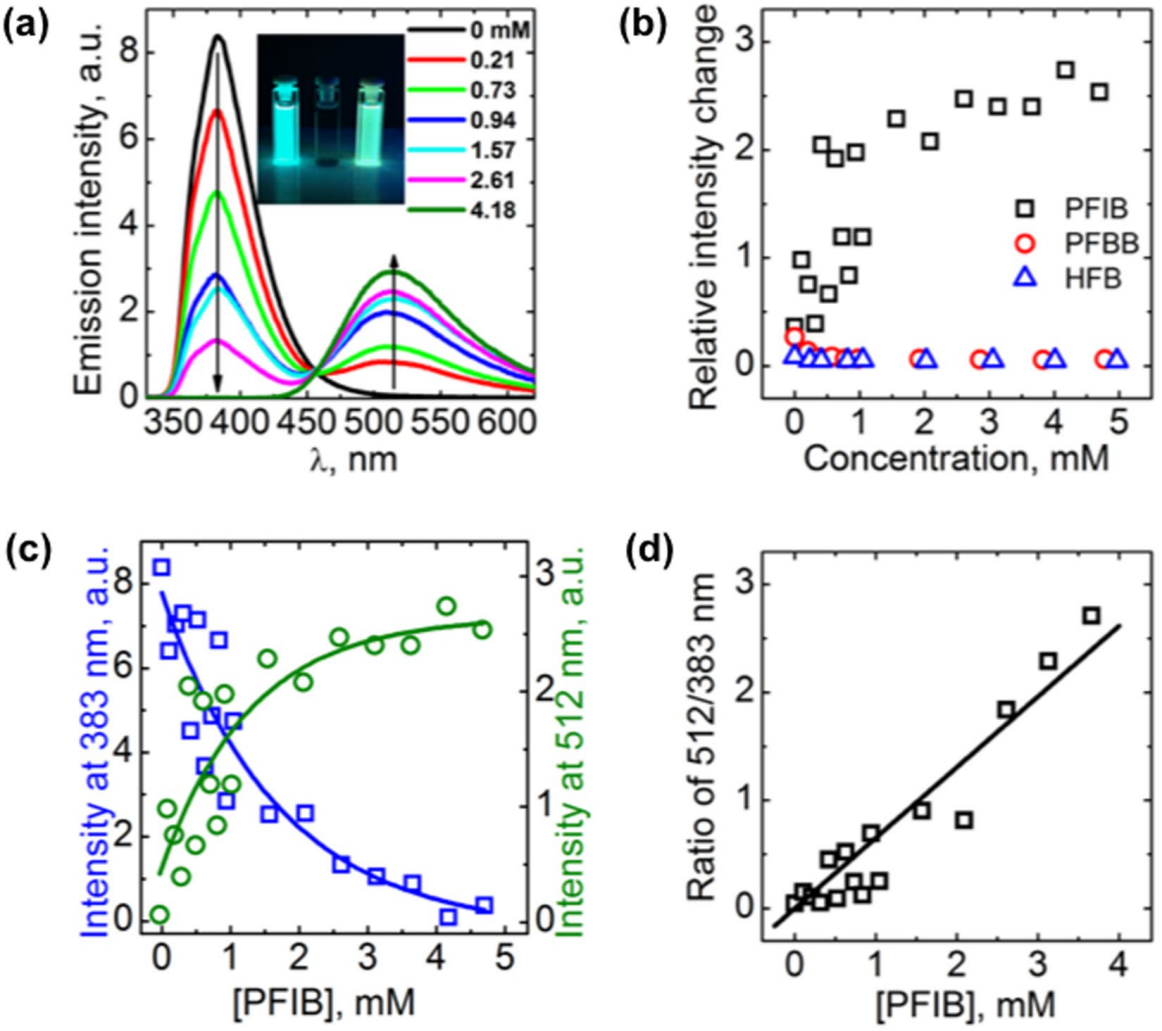
**(a)** Emission spectra of **A** (6.4 μM in DCM) upon titration with PFIB. Inset: photographs of the emission color of **A** (left), PFIB (center), and the **A**:PFIB mixture (right) under 365 nm illumination. **(b)** Emission intensity at 512 nm upon titration of **A** with PFIB, PFBB, and HFB. **(c)** Emission intensity of **A** upon titration with PFIB, monitored at 383 (blue squares) and 512 nm (green circles); **(d)** Emission intensity ratio between 512 nm and 383 nm.

Figure 2a presents the emission spectra of 6.4 μM DCM solution of **A** upon titration with PFIB (excitation wavelength 320 nm). The addition of PFIB results in notable spectral changes: the local excited state emission (383 nm) is quenched completely, while a new emission band centered at 512 nm appears, and the emission color turns from cyan to green (inset of Fig. 2a). In addition, a new absorption band at 385 nm is formed (Figure S4a,b), and is attributed to ground state complex formation. Excitation at this band leads to the 512 nm emission, increasing in intensity with respect to higher PFIB concentration (Figure S4c). When carrying out the titration in ACN, we observed 40% quenching of fluorescence at 380 nm with no enhancement of emission at 512 nm upon increasing the concentration of PFIB (Figure S5a). The lack of complex formation is attributed to the higher dielectric constant and polarity of ACN that increase the solute-solvent interactions, thereby hampering the halogen bond^47^.

To confirm that the spectral changes observed are induced by halogen bonding, we repeated the titration experiment with pentafluorobromobenzene (PFBB), a weaker halogen-bond donor, as well as hexafluorobenzene (HFB), which serves as a nonbonding reference. Neither PFBB nor HFB lead to fluorescence quenching at 383 nm band or emission enhancement at 512 nm (Fig. 2b) in the concentration range up to 5 mM. However, for higher PFBB concentrations (up to 100 mM) the quenching, complemented with minute increase of the fluorescence at 512 nm, does take place (presumably due to weak halogen bonding), while for HFB no 512 nm emission can be detected and only minor dynamic quenching^57^ (Figure S5b,c) is observed at 383 nm. These results unambiguously show that the observed fluorescence modulation of **A** is due to halogen bonding.

Being able to monitor both the concentration of unbound **A**, *c(unbound)*, from the 383 nm band, and the concentration of complexed **A**, *c(bound)*, from the intensity of the 512 nm band, allowed us to determine the binding constant *K*_*b*_ between **A** and PFIB [*Q*] from the ratio between those two fractions. Fitting the data shown in Fig. 2c yielded *K*_*b*_ of 650 ± 40 M^−1^, deduced from the equation below^42^:

1 $$\frac{c(bound)}{c(unbound)}={K}_{b}[Q]$$

Given that we are dealing with a mono-dentate neutral halogen-bonded system, this value can be considered very high, which can be at least partly attributed to the central carbazole core.

**Figure 3:**
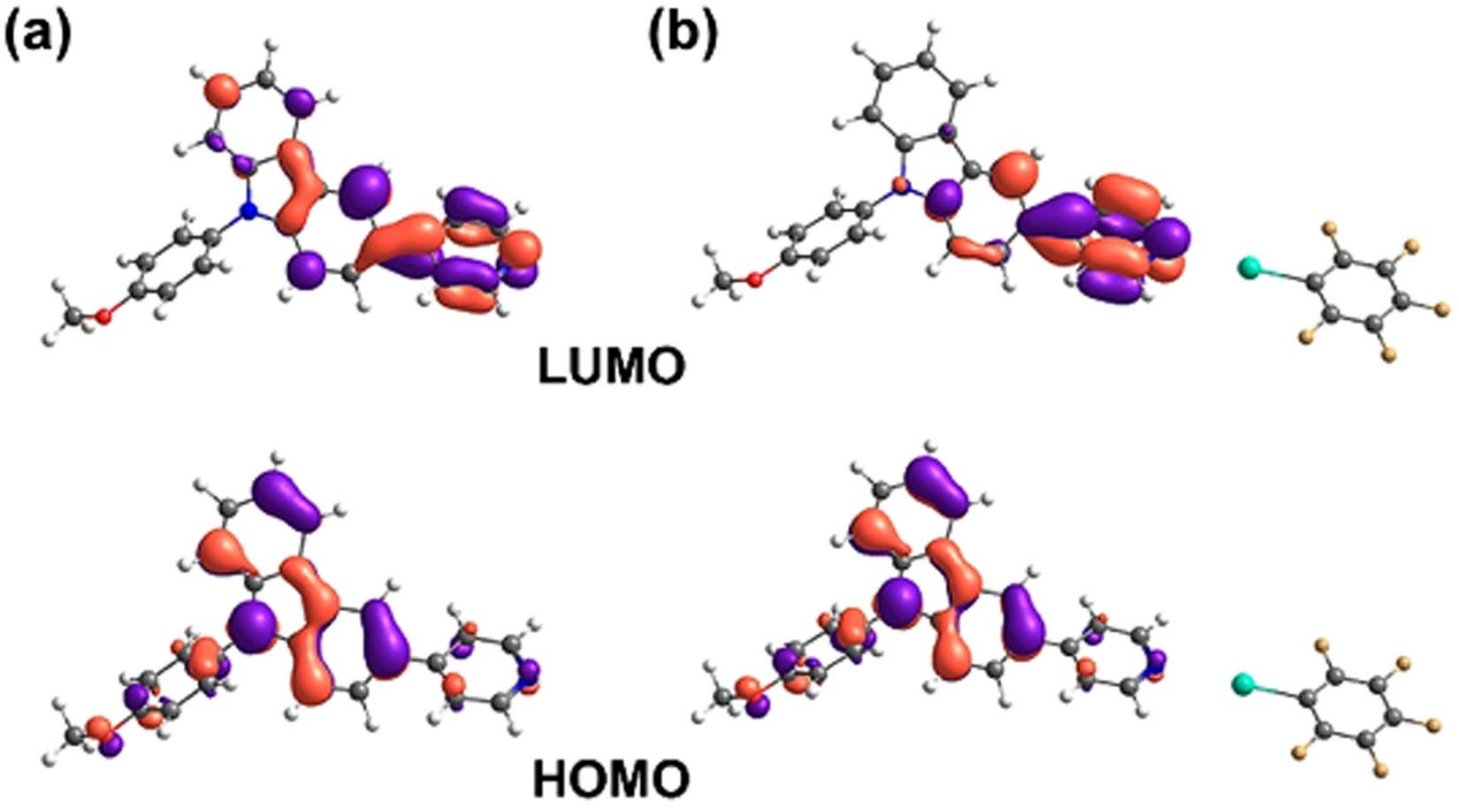
Optimized geometric structures and frontier molecular orbitals of **(a) A** and **(b) A**:PFIB complex, calculated with DFT at PBE1PBE/LANL2DZ/6-31 G** level of theory (isosurface value = 0.04).

To understand the nature of the fluorescence modulation presented in Fig. 2, we studied the frontier molecular orbitals of **A** and **A**:PFIB complex with DFT. As shown in Figs 3 and S7, for the highest occupied molecular orbital (HOMO) of **A** the electron density distributes mostly over the electron-rich carbazole unit, while for the lowest unoccupied molecular orbital (LUMO) the electron density is shifted towards pyridine. In the **A**:PFIB complex, HOMO remains unchanged as compared to **A**, however, significant changes occur in the LUMO and the electron density is almost completely localized on the pyridine moiety. A shortening of the C-C bond between the carbazole and pyridyl moieties (d_C-C_ = 1.476 Å in **A** vs d_C-C_ = 1.475 Å in **A**:PFIB) and a −0.081 charge transfer to PFIB (Mulliken population analysis) upon complexation was determined. Further analysis of the **A**:PFIB complex orbitals revealed that closest PFIB orbitals contribution are located in H-2 and L+3, relating to HOMO and LUMO of PFIB alone (Figure S7), respectively. This computational analysis indicates that the fluorescence modulation of **A**, induced by halogen bonding, arises through intramolecular charge transfer from carbazole to pyridine, similarly to what has been previously reported for hydrogen-bonded cocrystals^58^.

In order to investigate the changes in the excited-state relaxation mechanism as a result of XB-driven complex formation, we measured the fluorescence lifetimes upon titration of **A** with PFIB (Figure S6). Upon excitation at 300 nm the addition of PFIB reduced the emission lifetime of **A** at 380 nm from 3.8 ns to 3.2 ns. The intensity of the band decreased, as expected based on the steady-state fluorescence titration (Fig. 2a), due to the formation of the **A**:PFIB ground-state complex, with a simultaneous small decrease in the emission lifetime due to electron transfer (Figure S6a,c). When exciting **A**:PFIB at 375 nm and monitoring the emission at 520 nm, the decay curves are monoexponential up to 1 mM PFIB concentration and biexponential beyond that. The lifetimes of ≤0.12 ns and 4.28 ns remain constant at all PFIB concentrations, while the emission intensity systematically increases (Figure S6b,d). The relative amplitude of the ≤0.12 ns omponent increases with increasing PFIB concentration, reaching 12% at 4.5 mM. We attribute the fast component to the quenched decay of excited carbazole formed after direct excitation of the **A**:PFIB complex.

**Figure 4:**
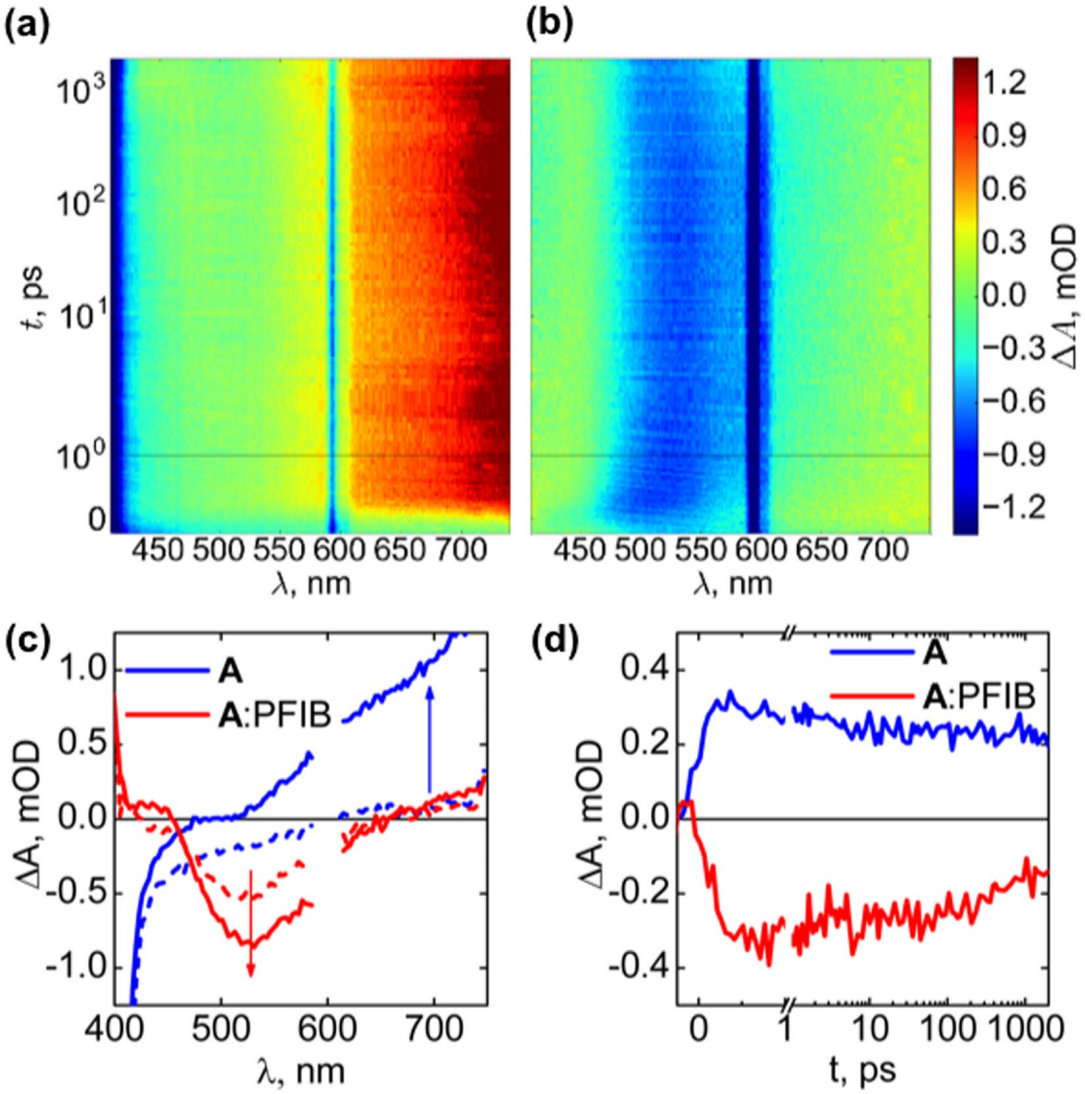
Ultrafast transient absorption spectra contour plots of (**a**) 50 µM **A** and (**b**) 50 µM **A** with 8 mM PFIB in DCM with 300 nm excitation; (**c)** Transient absorption spectra of **A** and **A**:PFIB before (dashed at −0.2 ps) and after excitation (solid at 1.0 ps); and (**d)** Transient absorption decays at 525 nm for **A** and **A**:PFIB, normalized to zero amplitude before excitation. All timescales are linear until 1 ps and logarithmic for longer delay times.

Ultrafast transient absorption spectroscopy (TAS) of the PFIB:**A** complex was performed to elaborate on the formation and excited state dynamics of the charge-transfer complex. The TAS spectrum of **A** alone shows the tail of the local excited state fluorescence at all delay times below 430 nm (Fig. 4a and c). The only transient feature in the spectrum is a wide positive absorption band located above 520 nm, assigned here to the transient absorption of the local excited state of the carbazole moiety. When PFIB (8 mM) is added into the 50 µM **A** solution the local excited state emission and transient absorption are quenched almost completely (Fig. 4b and d). The remaining small positive transient absorption signal above 650 nm is assigned to unbound **A**. A strong negative transient signal is observed between 460–630 nm, assigned to the emission from the **A**:PFIB complex. The emission is already observed at negative delay times, however, it increases in magnitude immediately after excitation (Fig. 4d). This means that the emissive charge-transfer state is formed directly after excitation at 300 nm, instead of being formed via electron transfer from the local excited state. Thus, we deduce that the charge-transfer complex is formed as a ground state complex, as also supported by DFT, that can be directly excited to the complex LUMO on a timescale faster than 1 ps.

## Conclusions

In summary, we have proposed an approach to tune the luminescence of conjugated semiconducting materials in solution, via halogen-bond-induced intramolecular charge transfer. The interaction is enhanced by the electron-rich carbazole core, which increases the electron density of the XB-accepting pyridine moiety. Thereby, pronounced emission color change can be observed even in solution upon complexation with XB-donating pentafluoroiodobenzene molecules. The main implications of this finding relate to halogen-bond-based “supramolecular electronics”, paving way for rational design of, e.g., tunable light emitting devices using simple and cost-effective organic semiconductors, and in our future efforts we will concentrate on utilizing the phenomenon in the solid state.

## Methods

### Synthesis

Anhydrous solvents and chemicals were purchased from Sigma-Aldrich and used as received. Spectroscopic grade dichloromethane (DCM), tetrahydrofuran (THF) and acetonitrile (ACN) were used for optical characterization. Synthesis of compound **1** has been carried out according to previously reported procedure^39^. The chemical reactions were carried out under argon or nitrogen environment. ^1^H and ^13^C NMR spectra were recorded with a Varian Mercury 300 MHz spectrometer (Varian Inc.) in DMSO-d_6_ against tetramethylsilane as reference. Mass spectroscopy was carried out using a high-resolution ESI-TOF LCT Premier XE mass spectrometer (Waters Corp.). The analyte was dissolved in chloroform:methanol (*c* ≈ 0.01 mg mL^−1^) and infused at a rate of 15 μL min^−1^. Purification of the products was carried out by column chromatography on silica gel 60 (Sigma Aldrich) with mesh size 0.040–0.063 mm.

### Steady-state spectroscopy

Absorption spectra were recorded using a Shimadzu UV-2501PC spectrophotometer. Steady-state fluorescence spectra were recorded with a Fluorolog Yobin Yvon-SPEX fluorometer. The excitation wavelengths were 300 and 320 nm, and the spectra were automatically corrected using a correction function provided by the manufacturer. Fluorescence quantum yields (Φ_f_) in solution were measured following a method using carbazole (Φ_f_ = 0.367 in DCM) as the ref.^59^. Dilute solutions (optical density at λ_ex_ < 0.1) of **A** in either THF or DCM was used for the fluorescence spectra recording in 1 cm path length cuvettes. Integrated areas of fluorescence spectra were used for further calculations by using equation (2):

2 $${\phi }_{f(X)}={\phi }_{f(R)}\frac{A{^{\prime} }_{(R)}{I}_{(X)}{n}_{(X)}^{2}}{A{^{\prime} }_{(X)}{I}_{(R)}{n}_{(R)}^{2}}$$

where subscripts *(R)* and *(X)* refer to reference compound and analyte, A’ is an optical density at the excitation wavelength, *I* is the area under the fluorescence spectrum, *n* is the refractive index of the medium.

### Time-resolved spectroscopy

The fluorescence decay curves were measured using a time-correlated single photon counting (TCSPC) system (PicoQuant GmBH) consisting of a PicoHarp 300 controller and a PDL 800-B driver. The samples were excited with either the pulsed diode laser head LDH-P-C-375 and (PicoQuant GmBH) at 375 ± 10 nm at a time resolution of 60 ps or sub-nanosecond pulsed LED source PLS 300 (PicoQuant GmBH) at 295 nm ± 5 nm at a time resolution of 600 ps. The signals were detected with a microchannel plate photomultiplier tube (Hamamatsu R2809U). Fluorescence decays were collected with a constant accumulation time. The instrumental response function (IRF) was measured separately, and the decays were simultaneously deconvoluted and fitted globally by applying the iterative least-squares method to the sum of exponents (eq. 3):

3 $$I(t,\lambda )=\,\sum _{i}{a}_{i}(\lambda ){e}^{-t/{\tau }_{i}}$$

where *τ*_*i*_ is the global lifetime, *α*_*i*_*(λ)* is the local amplitude (preexponential factor) at a specific wavelength.

The ultrafast transient absorption spectroscopy was studied using the pump-probe method. Fundamental laser pulses were generated with a Ti:Sapphire laser (Libra F, Coherent Inc., 800 nm, ~100 fs pulse width, repetition rate 1 kHz). About 90% of the fundamental beam energy was directed to an optical parametric amplifier (Topas C, Light Conversion Ltd.) to produce the excitation pump pulses (300 nm, approximately 0.5 mm beam diameter at the sample, attenuated to 0.3 µW per pulse at the sample). The remaining 10% of the fundamental laser energy was directed through a motorized translational stage (delay line) to a deionized water cuvette for white continuum generation of probe pulses. The probe light was split into two beams, reference and signal. The measurement system (ExciPro, CDP systems) was equipped with a Si CCD array detector for the visible part of the spectrum. A chopper synchronized with the fundamental laser pulses was used to block every second pump pulse, and the absorbance change was calculated from consecutive pulses. The measurements were averaged 2,000 times.

### Computational Details

All the details regarding computational analysis given in supporting information.

## Electronic supplementary material

Supplementary Information

## Data Availability

The data that support the findings of this study are available from the corresponding author upon request.
