# Supplementary material for: Halogen-Bond-Assisted Photoluminescence Modulation in Carbazole-Based Emitter
Source: Sci Rep. 2018 Sep 26;8:14431. doi: 10.1038/s41598-018-32830-3 (PMC6158238; doi:10.1038/s41598-018-32830-3)
Supplement: Supplementary file 1 — Supplementary Information [file 41598_2018_32830_MOESM1_ESM.docx]

**Supporting Information**

**for**

Halogen-Bond-Assisted Photoluminescence Modulation in Carbazole-Based Emitter

Jagadish K. Salunke^‡^, Nikita A. Durandin^‡^, Tero-Petri Ruoko, Nuno R. Candeias, Paola Vivo, Elina Vuorimaa-Laukkanen, Timo Laaksonen, Arri Priimagi*

Laboratory of Chemistry and Bioengineering, Tampere University of Technology, P.O. Box 541, FI-33101 Tampere, Finland.

Synthesis of **A**

Synthesis of *N*-(4-methoxyphenyl) carbazole was carried out by using Ullman-coupling reaction of commercially available carbazole with 4-iodoanisole. It was further mono-brominated using *N*-bromosuccinimide (NBS) at room temperature. The halogen-bond acceptor **A** was synthesized by using Suzuki-cross coupling reaction of 3-bromo-*N*-(4-methoxyphenyl) carbazole with 4-pyridineboronic acid pinacol ester (Scheme S1).


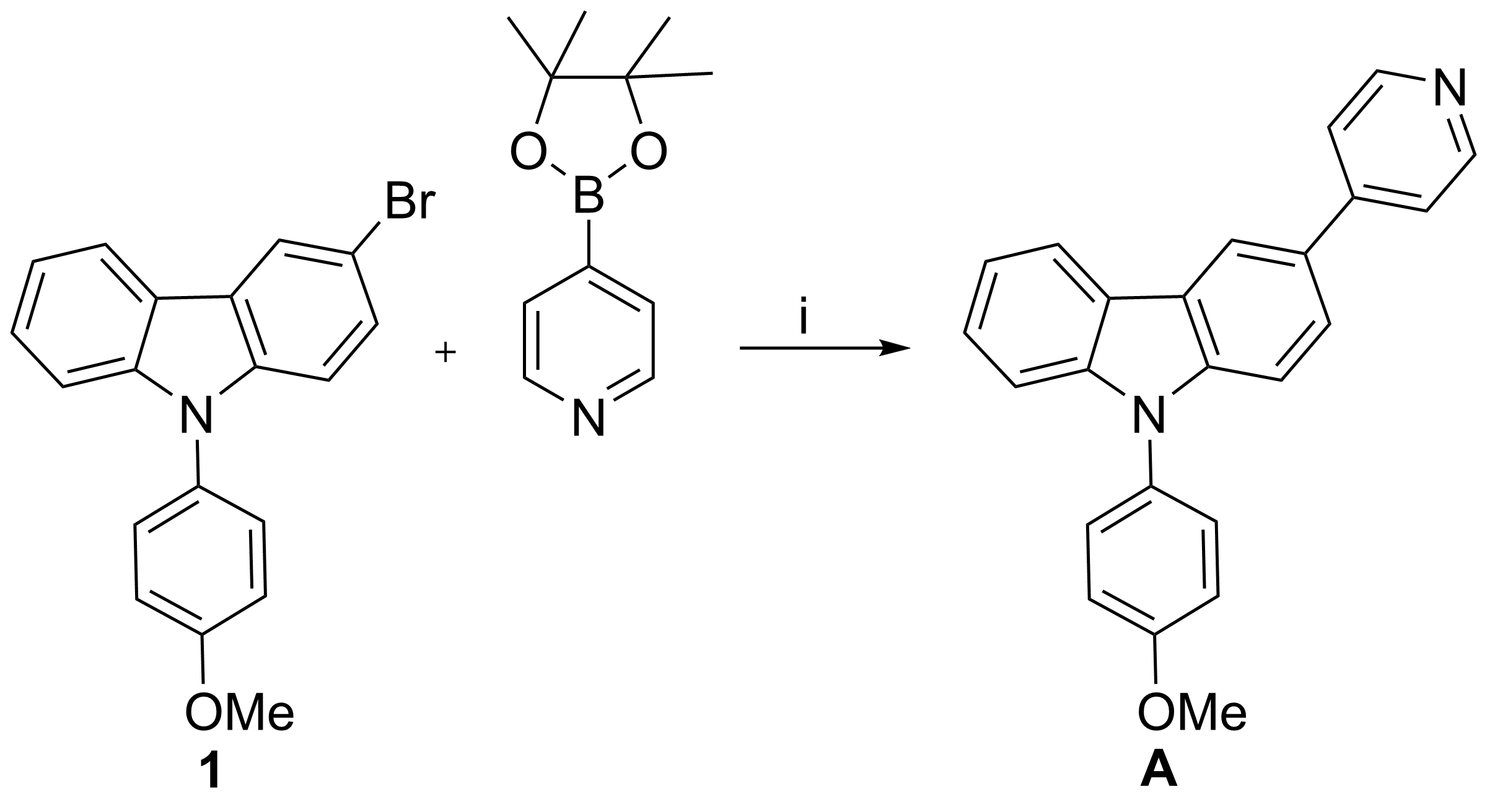


Scheme S1. Synthesis of **A**

9-(4-methoxyphenyl)-3-(pyridin-4-yl)-9H-carbazole (**A**). 3-Bromo-*N*-(4-methoxyphenyl) carbazole (2 g, 4.6 mmol), 4-pyridineboronic acid pinacol ester (1.037 g, 5.06 mmol), 2 M aqueous K_2_CO_3_ and 25 ml of THF:Toluene mixture (20:5) were added into a Schlenk flask. After degassing for 30 min by purging with nitrogen, the catalyst Pd(PPh_3_)_4_ (0.531 g, 0.46 mmol) was added to the reaction mixture and stirred for 24 hr in reflux. Following confirmation of reaction completion by TLC, the reaction mixture was extracted using dichloromethane (200 ml) and water, and dried over sodium sulfate (Na_2_SO_4_). After evaporation of the solvent under vacuum, the crude product was purified by silica gel column chromatography DCM:methanol (95:5) mixture as eluent to afford 1.2 g of a green solid in 70% yield. ^1^H NMR (300 MHz, DMSO-d_6_)δ= 8.77 (s, 1 H), 8.63 (d, *J*= 4.7 Hz, 2 H), 8.36 (d, *J* = 8.8 Hz, 1 H), 7.98 - 7.76 (m, 3 H), 7.59 - 7.41 (m, 3 H), 7.40 - 7.27 (m, 3 H), 7.26 - 7.17 (m, 2 H), 3.88 (s, 3 H); ^13^C NMR (75 MHz ,DMSO-d_6_) δ = 159.1, 150.6, 148.0, 141.6, 129.5, 129.2, 128.6, 127.1, 125.4, 123.7, 123.0, 121.4, 120.6, 119.5, 115.8, 110.4, 55.9. HRMS (m/z, ES+) calculated for C_24_H_18_N_2_O 351,1497, found 351,1534.


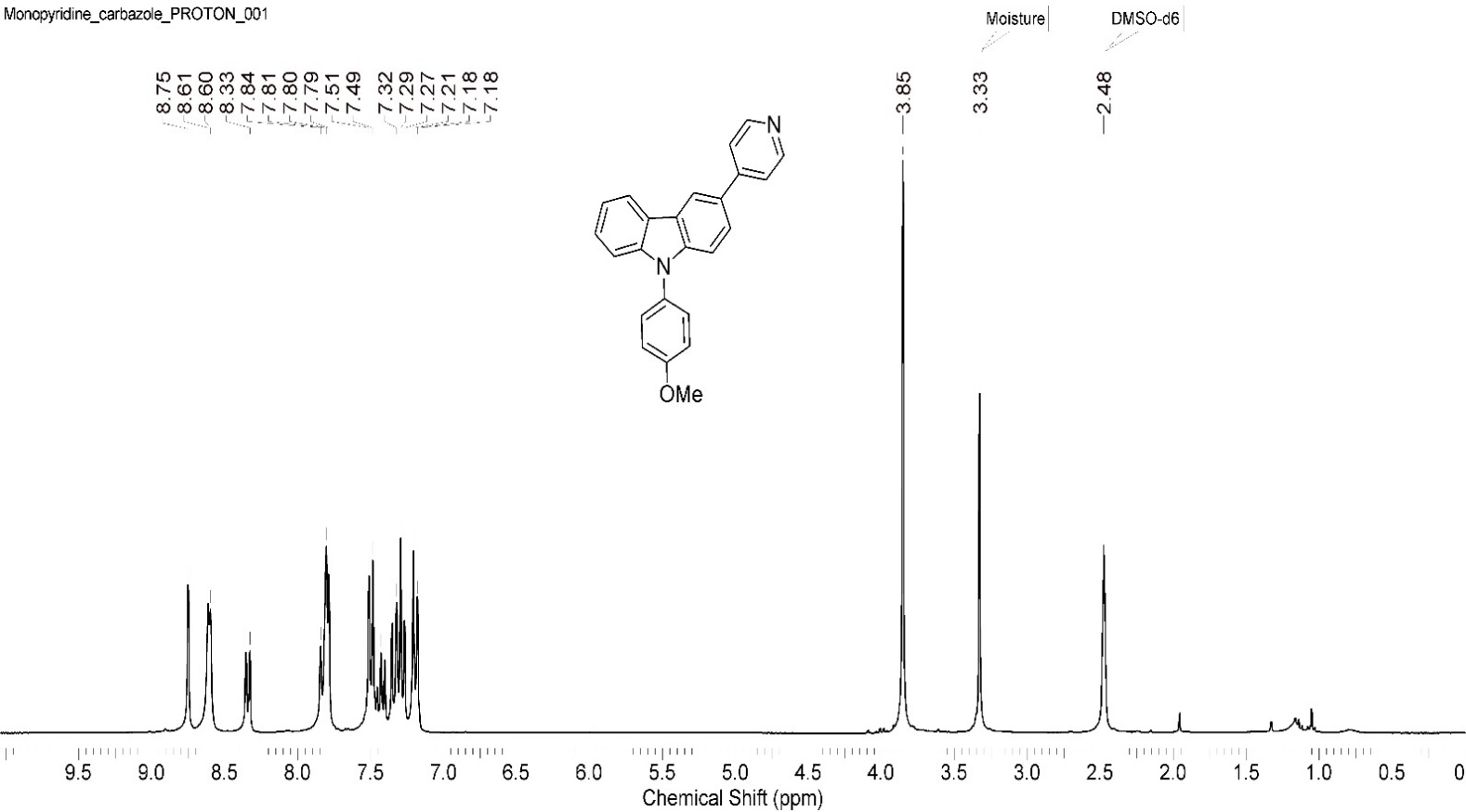


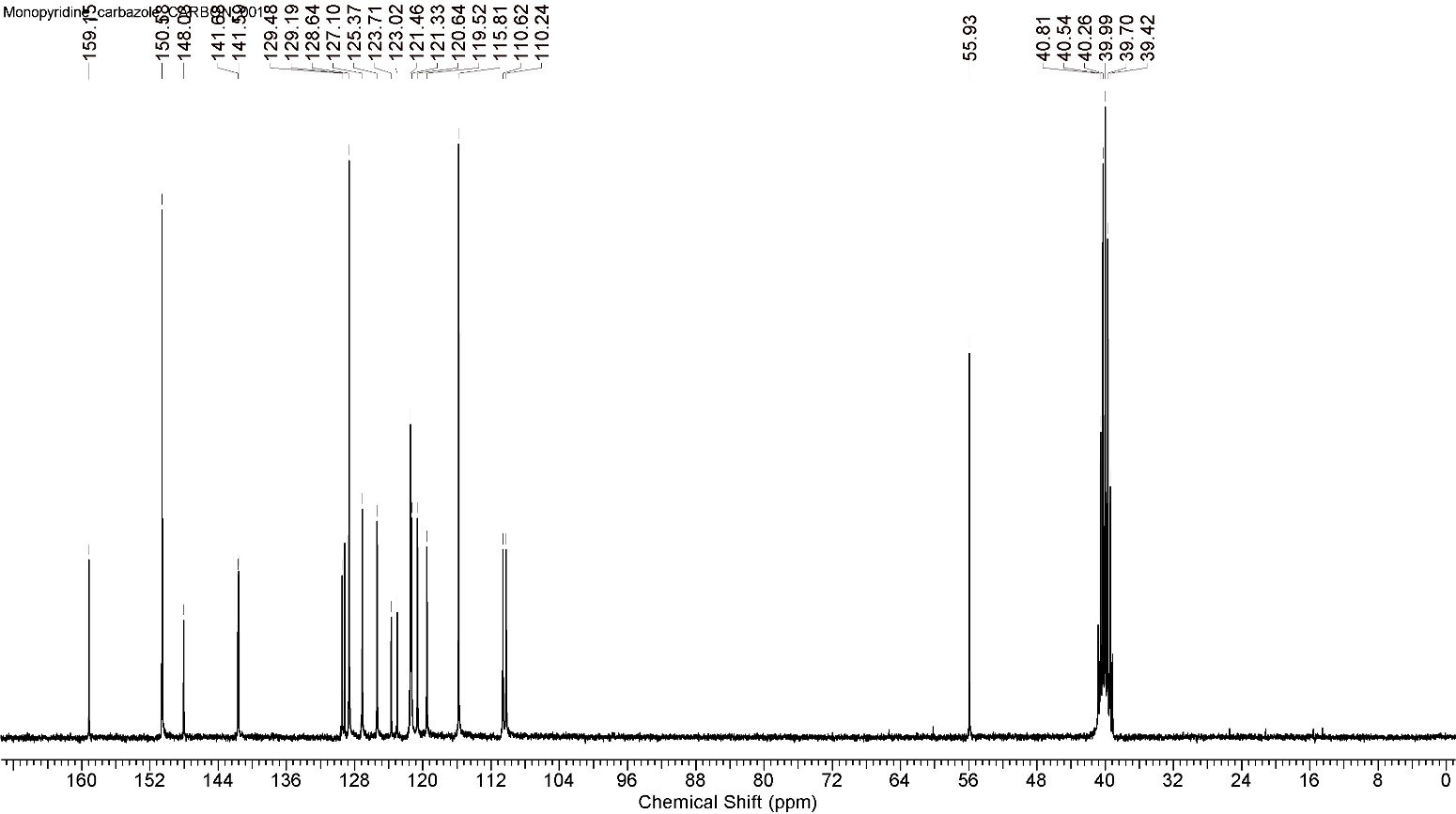


Figure S1. ^1^H and ^13^C NMR spectra of **A**


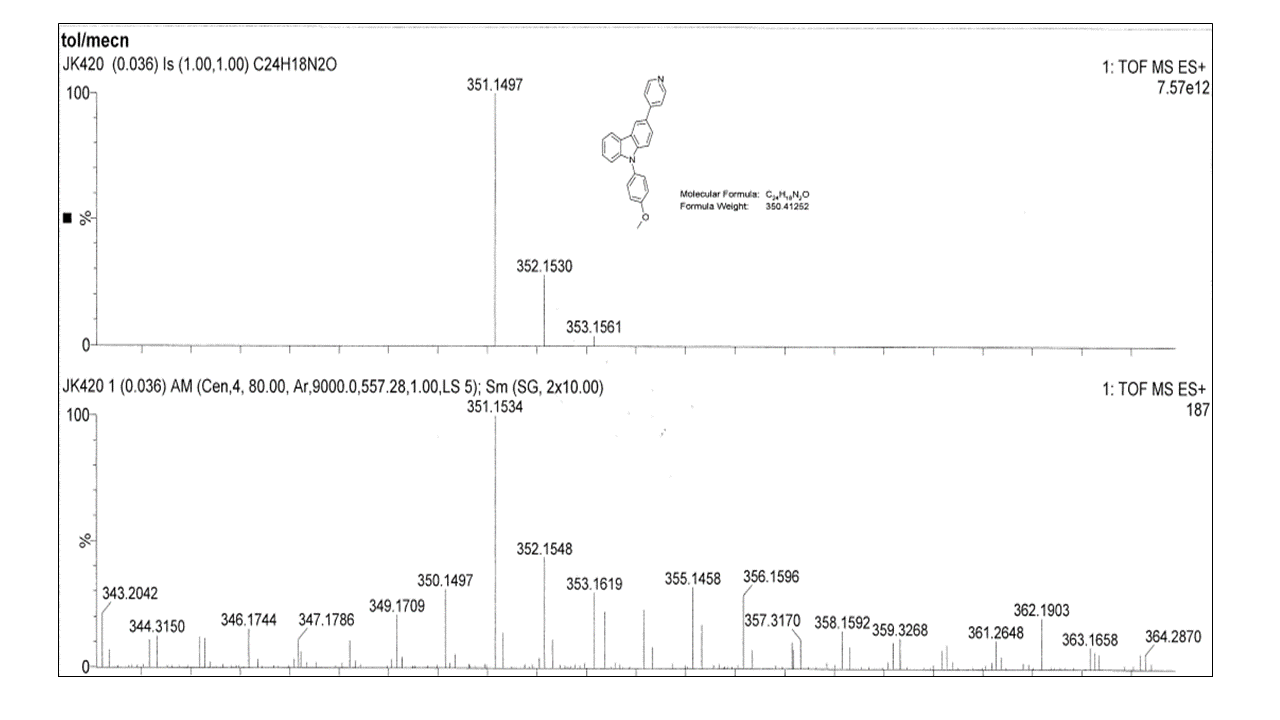


Figure S2. Mass spectra of **A**


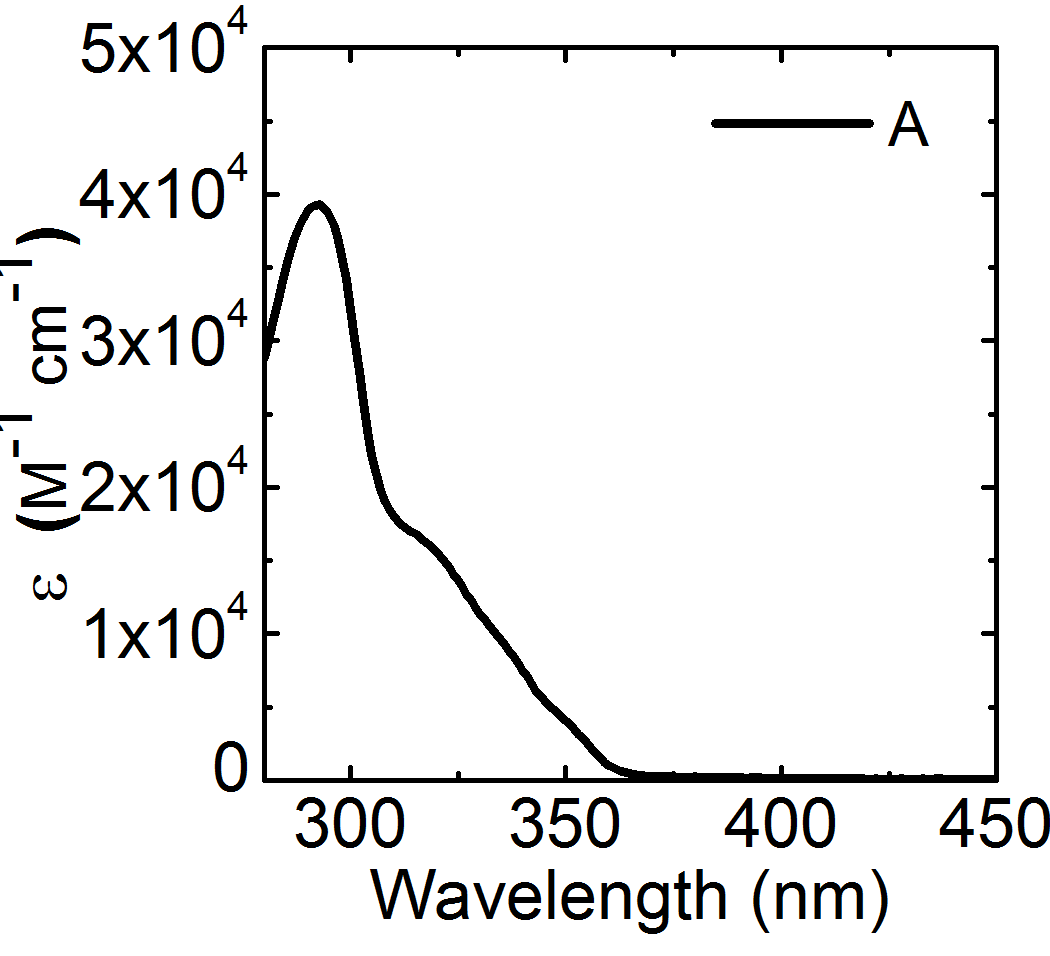


Figure S3. Molar extinction coefficient of A in DCM.


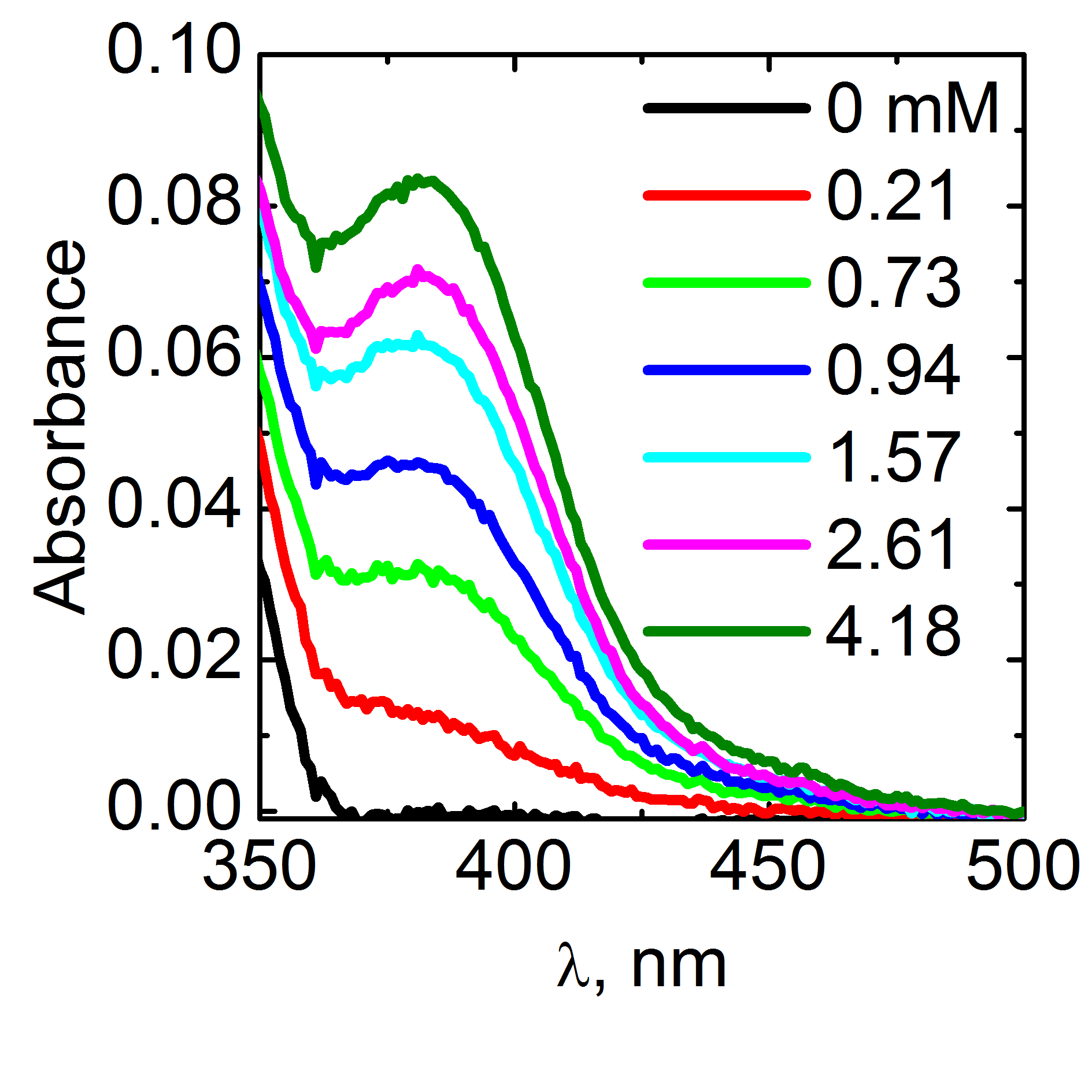


**b)**

**a)**


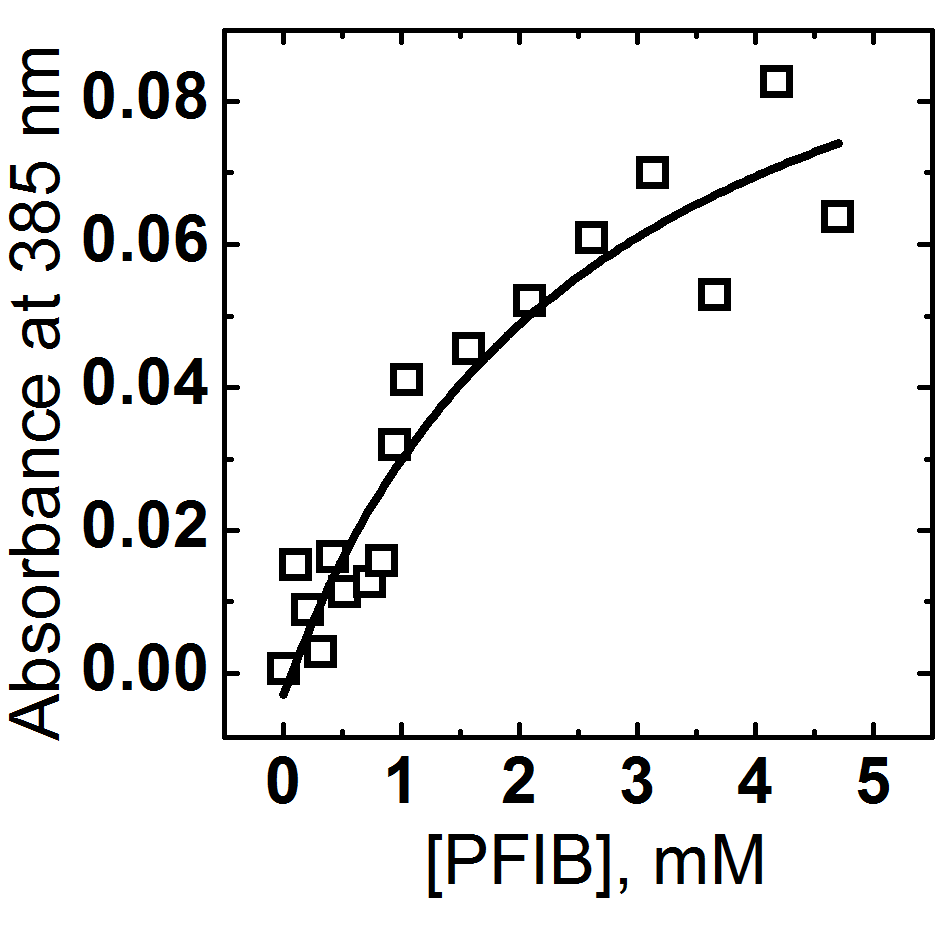


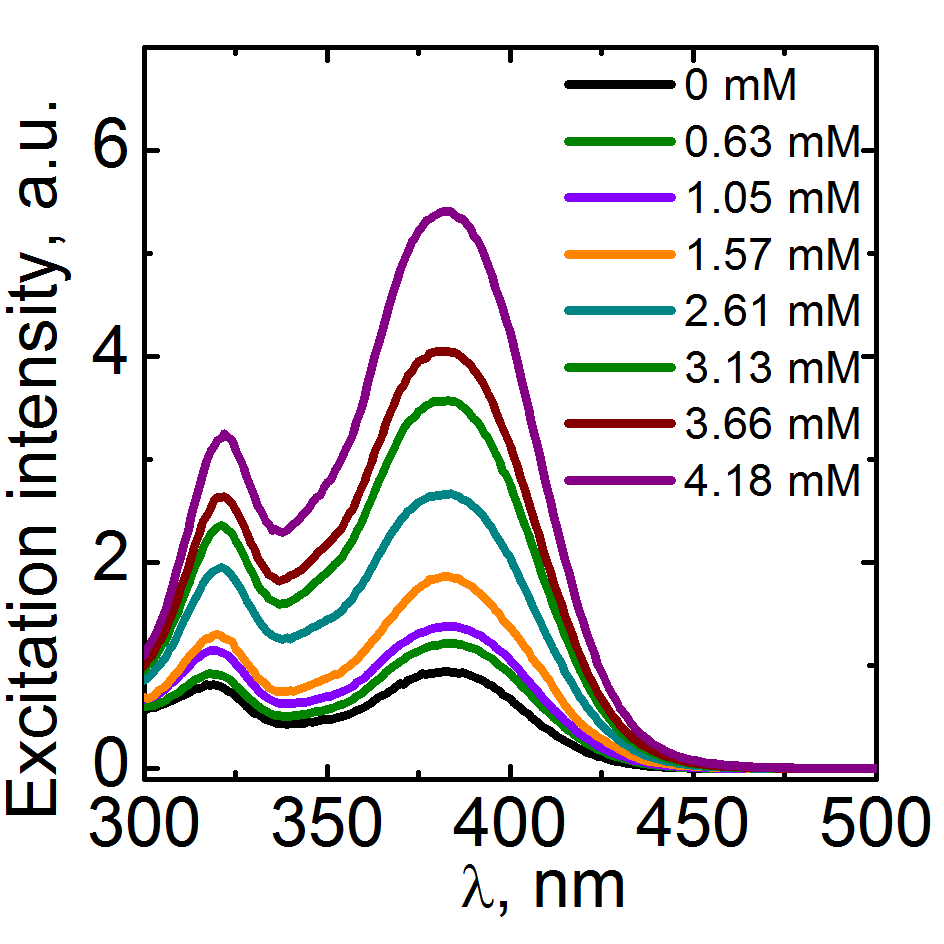

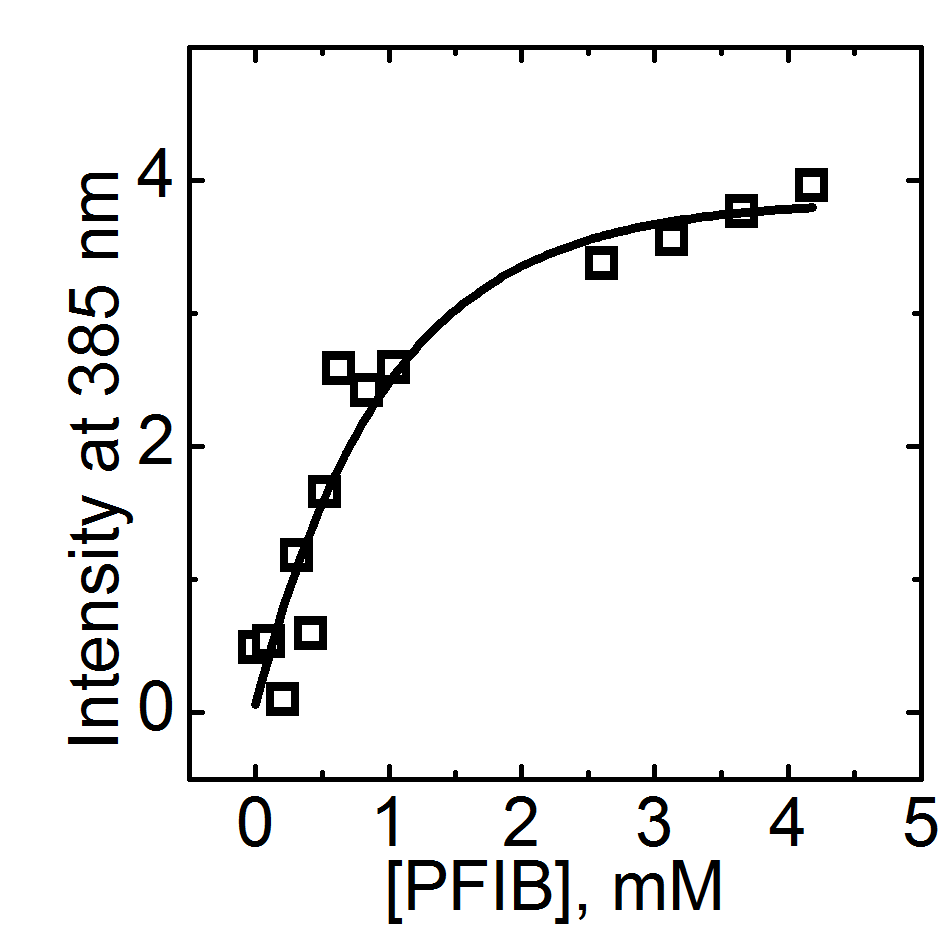

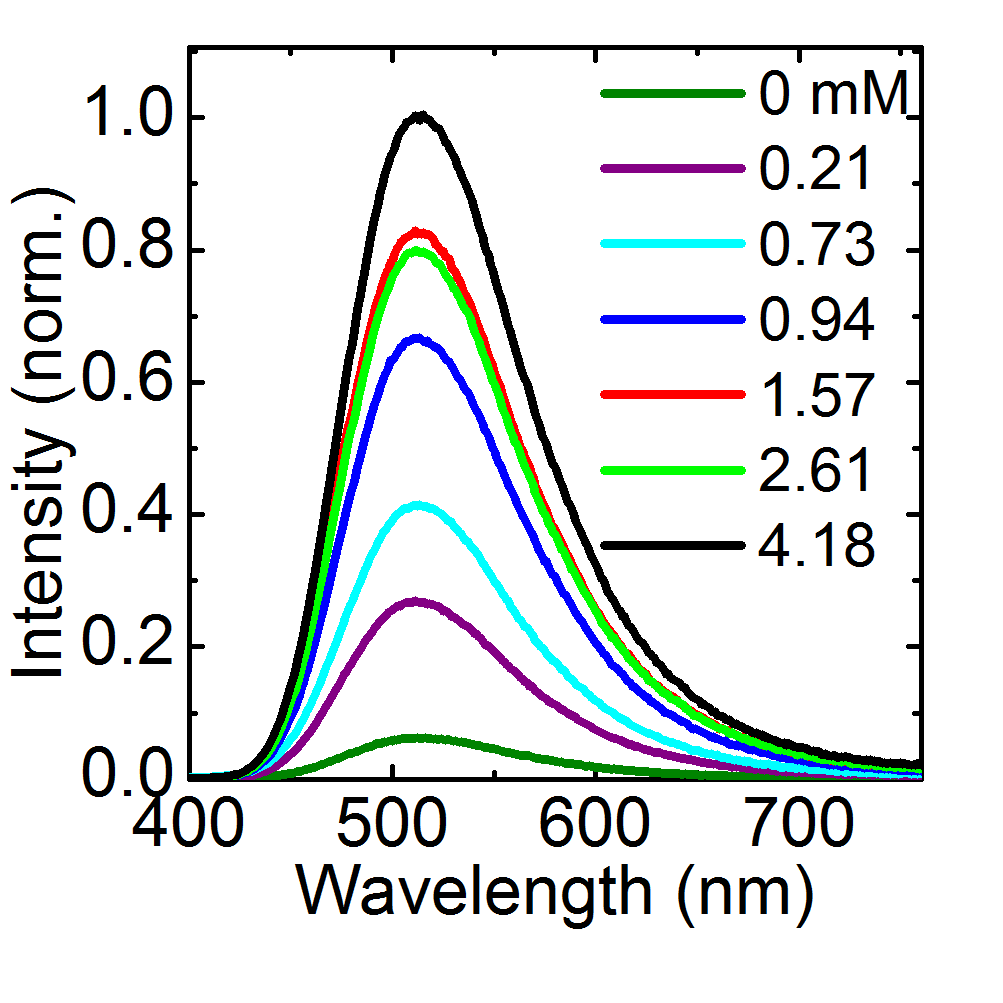


**d)**

**c)**

**e)**

Figure S4. (a) Absorption spectra of **A** (6.4 µM) with respect to the addition of PFIB in DCM. (b) Absorbances at 385 nm upon titration of **A** (6.4 µM) with different PFIB concentrations in DCM. (c) Excitation spectra of **A** (6.4 µM) with respect to the addition of PFIB in DCM. (d) Excitation intensity at 385 nm upon titration of **A** (6.4 µM) with different PFIB concentrations in DCM. (e) Emission spectra of **A** (6.4 µM) in presence of different concentrations of PFIB in DCM upon excitation at 385 nm.


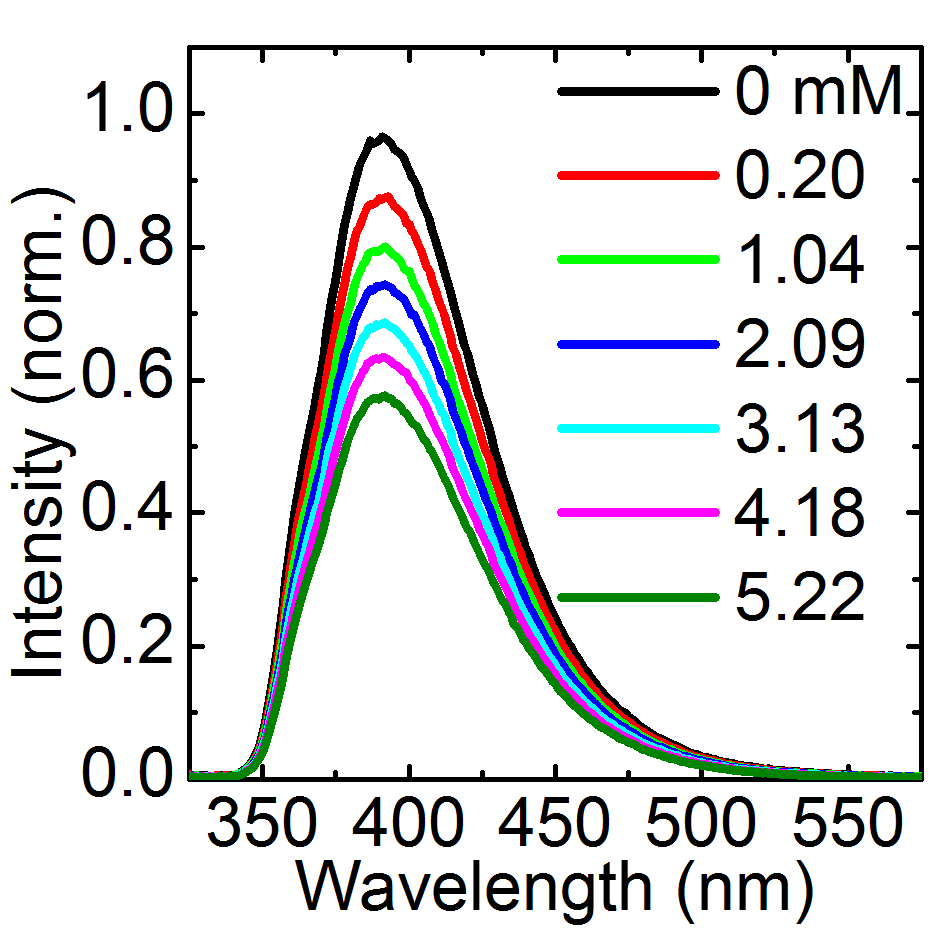

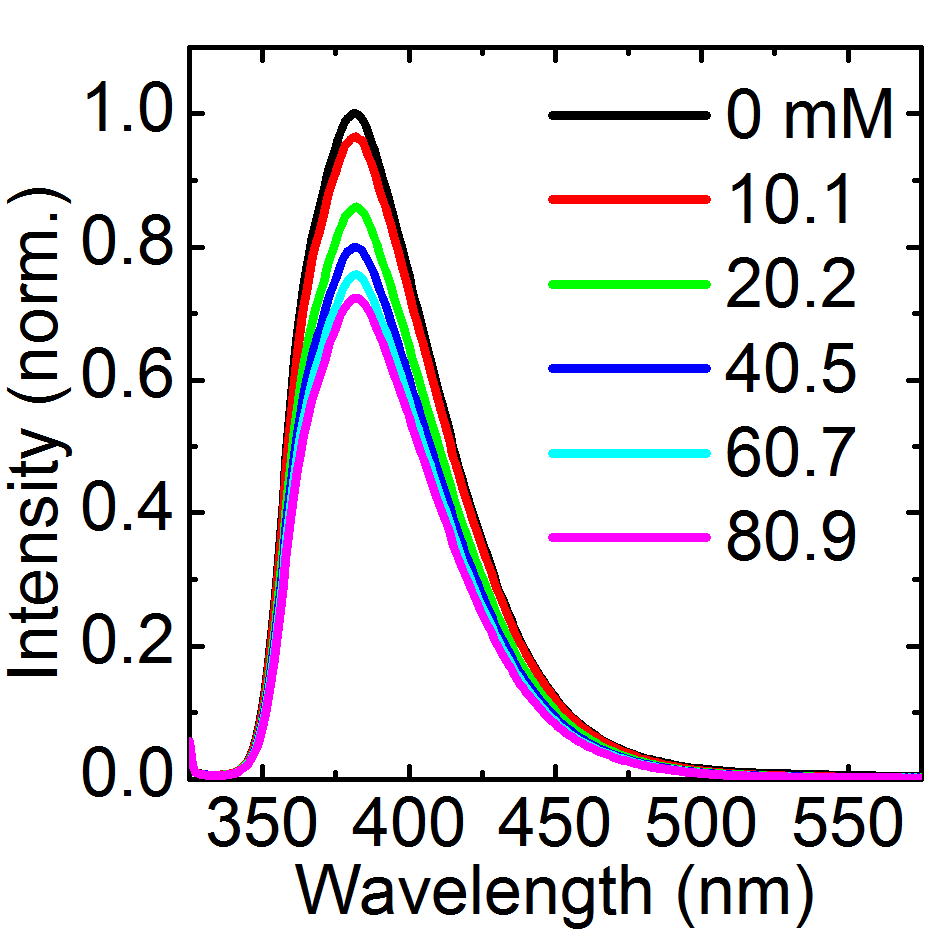


**a)**

**b)**


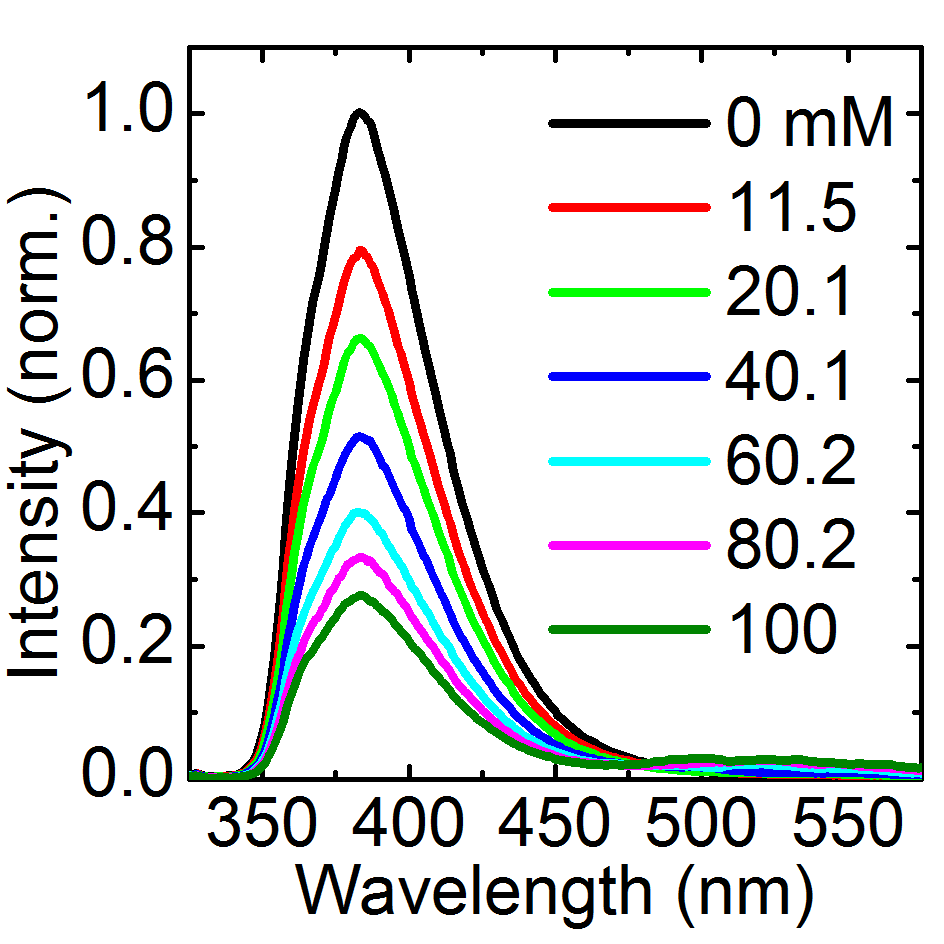

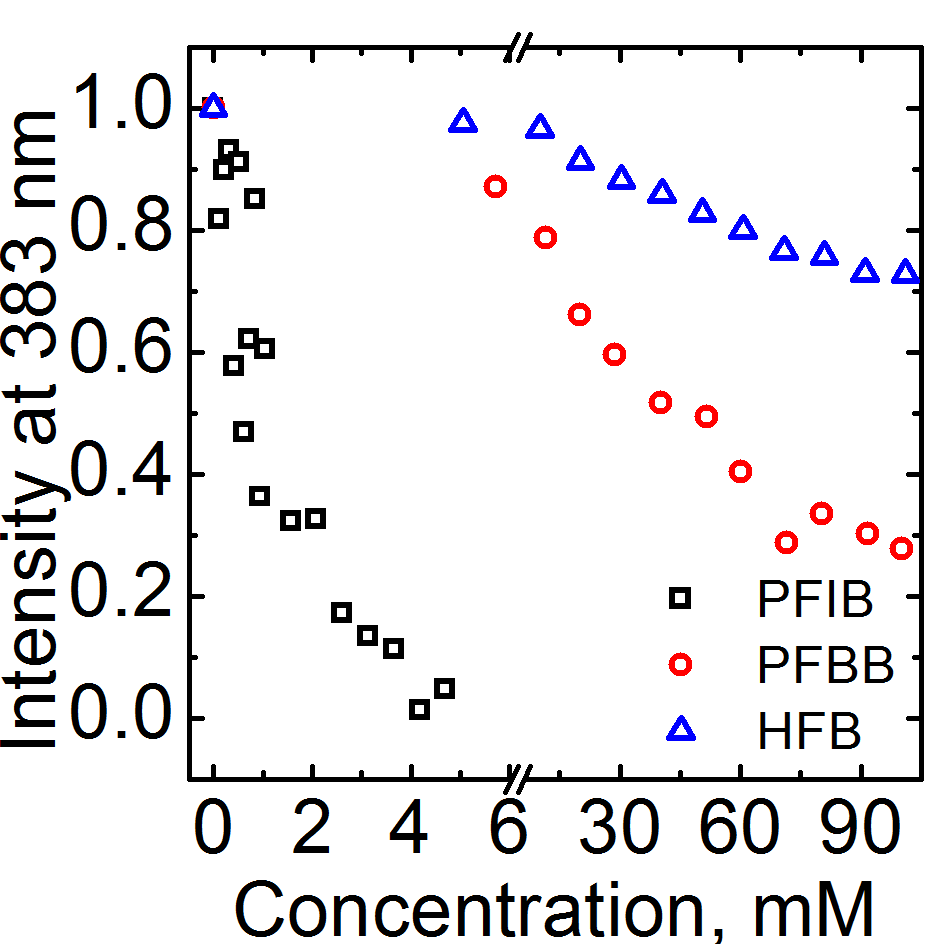

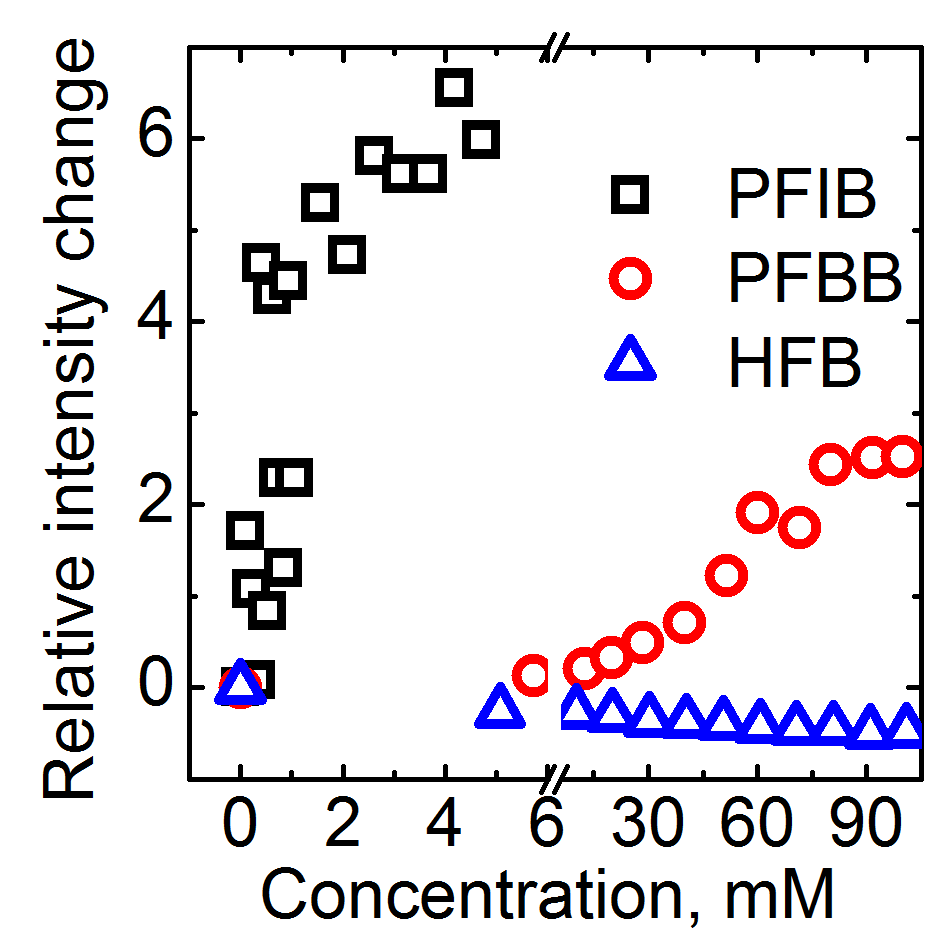


**e)**

**d)**

**c)**

Figure S5. (a) Emission spectra of **A**:PFIB mixtures with 6.4 µM of **A** and different PFIB concentrations in ACN. (b) Fluorescence spectra of 6.4 µM **A** in the presence of different HFB concentrations in DCM. (c) Emission spectra of A:PFBB mixtures with 6.4 µM of **A** and different PFBB concentrations in DCM. (d) Fluorescence titration curves of **A** with different XB donors monitored at 383 nm. (e) Relative change in emission intensity at 512 nm upon titration of **A** with PFIB, PFBB, and HFB (change of scale at 6 mM concentration).


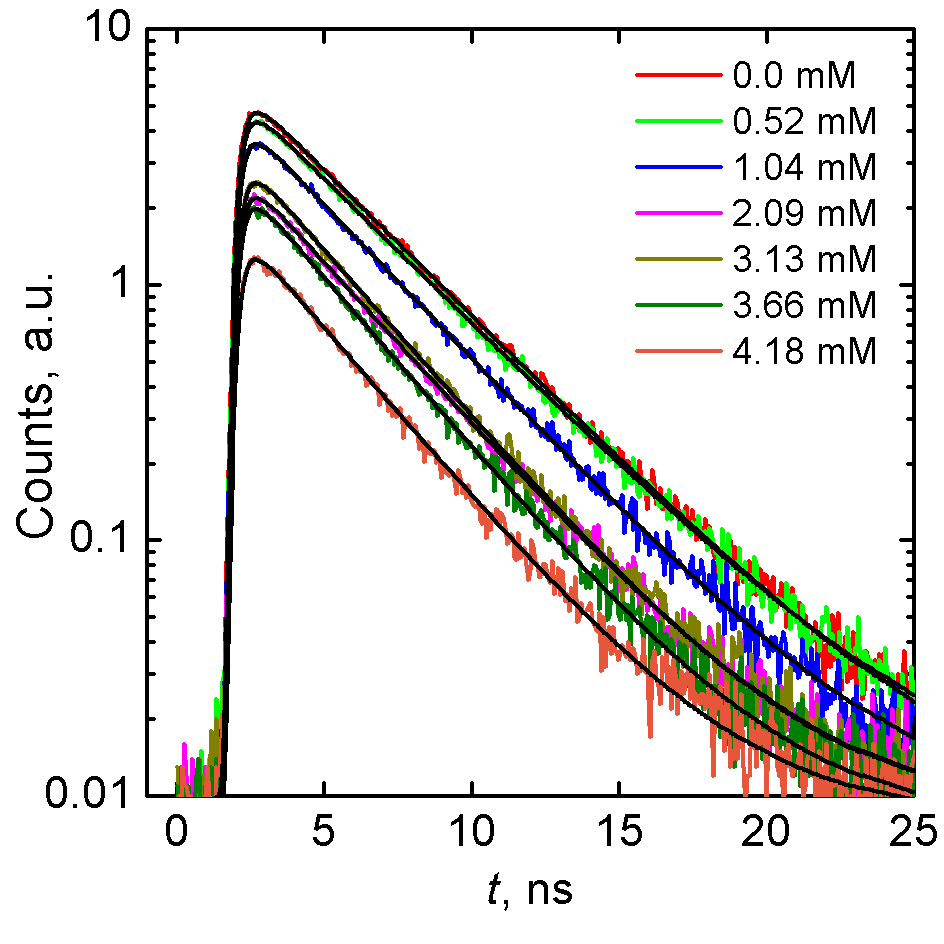

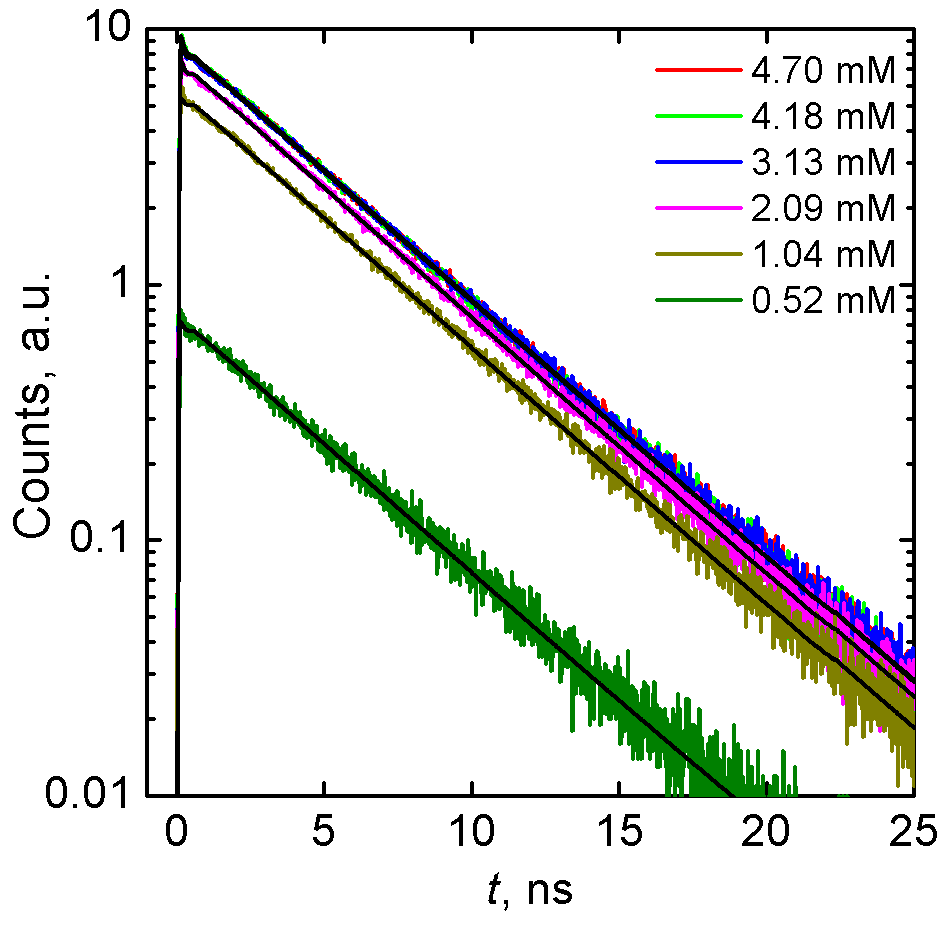

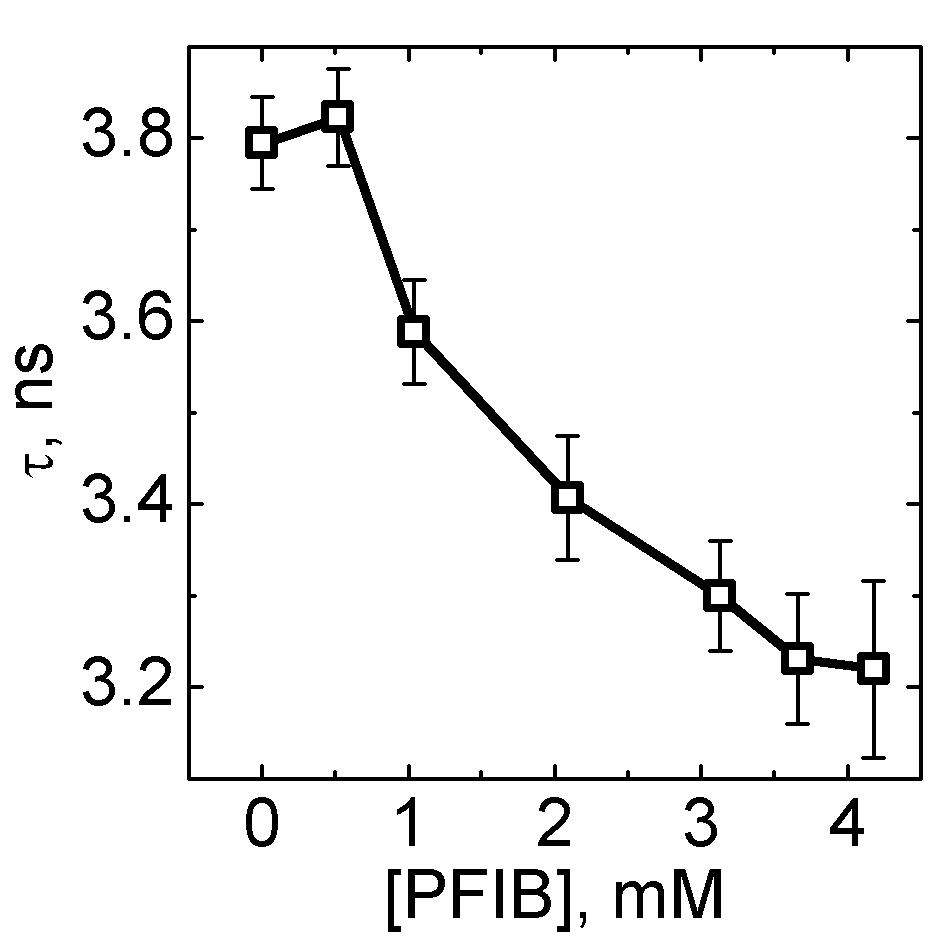

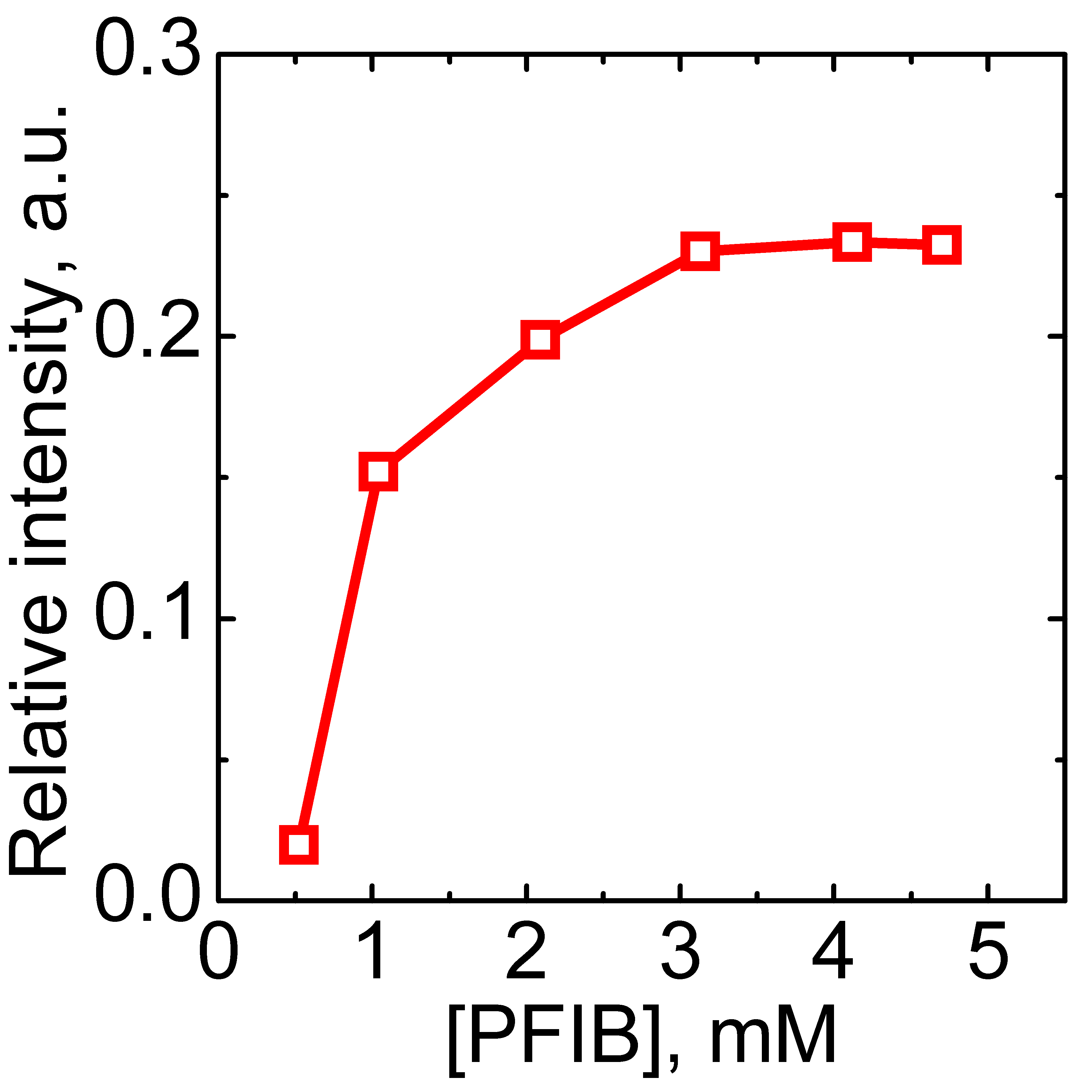


**Figure S6.** Fluorescence decay curves with (a) 300 nm excitation and 385 nm monitoring, and (b) 375 nm excitation and 515 nm monitoring of 6.4 µM **A** with different concentrations of PFIB in DCM. **(**c) Fluorescence lifetimes monitored at 380 nm with 300 nm excitation of 6.4 μM **A** in DCM; **(**d) Fluorescence lifetime intensities of the slower 4.28 ns component monitored at 515 nm with 375 nm excitation of 6.4 μM **A** in DCM.

**Computational Details**

All calculations were carried out using the Gaussian16^3^ software package without symmetry constrains in gas phase. Density Functional Theory (DFT)^4^ and Time-Dependent DFT (TD-DFT)^5^ were used for computation of the ground state and vertical excitations (considering the 15 lower lying transitions) of **A**, and **A**:PFIB. All calculations have been performed using the PBE1PBE functional and 6-31G(d, p)^6-10^ basis set for all atoms except iodide for which LANL2DZ^11-14^ was used. That functional uses a hybrid generalized gradient approximation (GGA), including 25% mixture of Hartree-Fock^15^ exchange with DFT exchange-correlation, given by Perdew, Burke and Ernzerhof functional (PBE).^16,17^ The basis-set superposition error^18^ was corrected for all calculations of complexes by using the counterpoise method as implemented in Gaussian16. The geometries of the complexes were optimized by calculations at the counterpoise-corrected PBE1PBE/LANL2DZ/6-31G(d, p) level in gas phase. Frequency calculations were performed to confirm the nature of the stationary points, yielding none imaginary frequency for the optimized geometries. Single point calculations were performed at the same level of theory, considering solvent effects (dichloromethane) using the Polarizable Continuum Model (PCM) initially devised by Tomasi and coworkers^22-29^, as implemented on Gaussian16. Natural charges of the optimized geometries were determined from a natural bond orbital analysis (NBO 3.1)^19-26^ as implemented in Gaussian16 taking solvent into consideration with the same model.

**Table S1:** Calculated gas-phase energies of interaction (E_int_) and halogen bond distance of optimized complexes of PFIB with pyridine and A at PBE1PBE/LANL2DZ/6-31G(d,p) level.

| **Complex** | **E_int_ (kcal/mol)** | **N••••I distance (Å)** |
| --- | --- | --- |
| Pyridine:PFIB | -5.78 | 2.895 |
| **A**:PFIB | -6.30 | 2.868 |

**Table S2**: Electronic excitation energies (eV), oscillator strengths (*f*) and configurations of the 5 low-lying excited states of **A**. Calculated by TDDFT//PBE1PBE/LANL2DZ/6-31G(d,p), based on single point calculation in DCM of the optimized ground state geometries in gas phase.

| **Electronic**  **Transition** | **Excitation Energy** | ***f****^a^* | **Composition***^b^* | **CI***^c^* | |
| --- | --- | --- | --- | --- | --- |
| S_0_ → S_1_ | 3.93 eV (315 nm) | 0.07 | H → L | 0.49769 | (50%) |
|  |  |  | H → L+1 | 0.46397 | (43%) |
|  |  |  | H-1 → L | 0.12381 | (3%) |
| S_0_ → S_2_ | 4.05 eV (306 nm) | 0.43 | H → L+1 | 0.48178 | (46%) |
|  |  |  | H → L | 0.47677 | (45%) |
|  |  |  | H-1 → L | 0.13466 | (4%) |
| S_0_ → S_3_ | 4.52 eV (274 nm) | 0.60 | H-1 → L | 0.62363 | (78%) |
|  |  |  | H-1 → L+1 | 0.21209 | (9%) |
|  |  |  | H → L+1 | 0.16948 | (6%) |
| S_0_ → S_4_ | 4.60 eV (269 nm) | 0.03 | H → L+2 | 0.68919 | (95%) |
|  |  |  | H-6 → L+4 | 0.1076 | (2%) |
| S_0_ → S_5_ | 4.72 eV (263 nm) | 0.15 | H-1 → L+1 | 0.51275 | (53%) |
|  |  |  | H → L+4 | 0.36607 | (27%) |
|  |  |  | H-1 → L | 0.2039 | (8%) |
|  |  |  | H → L+5 | 0.12719 | (3%) |
|  |  |  | H-2 → L+1 | 0.11211 | (3%) |
| ^a^Oscillator strength; ^b^H stands for HOMO and L stands for LUMO; ^c^Absolute CI coefficient of the wavefunction for each excitation. In parenthesis are indicated the percentage contribution of the configuration to excitation. | | | | | |

**Table S3**: Electronic excitation energies (eV), oscillator strengths (*f*) and configurations of the 5 low-lying excited states of **A**:PFIB. Calculated by TDDFT//PBE1PBE/LANL2DZ/6-31G(d,p), based on single point calculation in DCM of the optimized ground state geometries in gas phase.

| **Electronic**  **Transition** | **Excitation Energy** | ***f****^a^* | **Composition***^b^* | **CI***^c^* | |
| --- | --- | --- | --- | --- | --- |
| S_0_ → S_1_ | 3.88 eV (320 nm) | 0.38 | H → L | 0.64115 | (82%) |
|  |  |  | H → L+1 | 0.24963 | (12%) |
| S_0_ → S_2_ | 3.98 eV (311 nm) | 0.28 | H → L+1 | 0.61169 | (75%) |
|  |  |  | H → L | 0.25926 | (13%) |
|  |  |  | H-1 → L | 0.18582 | (7%) |
| S_0_ → S_3_ | 4.44 eV (279 nm) | 0.57 | H-1 → L | 0.62963 | (79%) |
|  |  |  | H-1 → L+1 | 0.2002 | (8%) |
|  |  |  | H → L+1 | 0.19153 | (7%) |
| S_0_ → S_4_ | 4.62 eV (268 nm) | 0.03 | H → L+5 | 0.66507 | (88%) |
|  |  |  | H → L+4 | 0.18061 | (7%) |
|  |  |  | H-6 → L+7 | 0.11045 | (2%) |
| S_0_ → S_5_ | 4.69 eV (264 nm) | 0.32 | H-1 → L+1 | 0.56642 | (64%) |
|  |  |  | H → L+7 | 0.2798 | (16%) |
|  |  |  | H-1 → L | 0.18681 | (7%) |
|  |  |  | H → L+8 | 0.15114 | (5%) |
| ^a^Oscillator strength; ^b^H stands for HOMO and L stands for LUMO; ^c^Absolute CI coefficient of the wavefunction for each excitation. In parenthesis are indicated the percentage contribution of the configuration to excitation. | | | | | |

**
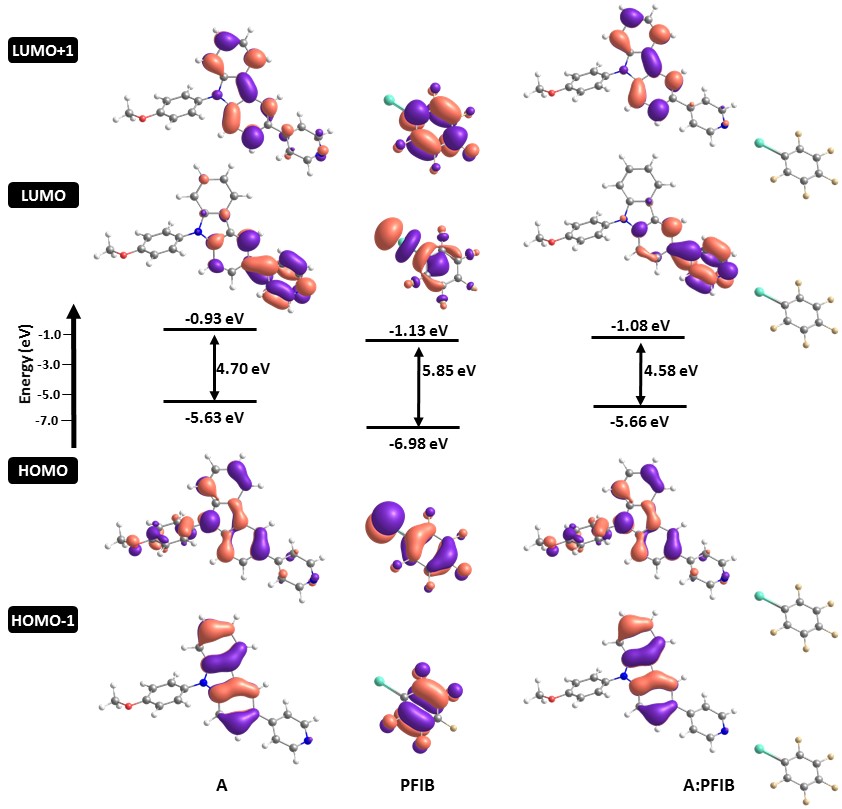
**

**Figure S7**. Optimized geometries in gas phase, selected molecular orbitals and frontier molecular orbitals energies of **A,** C_6_F_5_I **(PFIB)** and **A**:PFIB complex, calculated with DFT at PBE1PBE/LANL2DZ/6-31G** level of theory in DCM (isosurface value = 0.04).

Atomic coordinates for all the optimized species (PBE1PBE/LANL2DZ/6-31G**)

**C_6_F_5_I (PFIB)**

6 -3.193399 1.010915 -0.267956

6 -2.655970 -0.228081 0.062938

6 -3.501004 -1.293914 0.345524

6 -4.878501 -1.120234 0.297094

6 -5.407075 0.122288 -0.034711

6 -4.571082 1.197174 -0.319721

53 -5.374256 3.066835 -0.818873

9 -2.356883 2.004861 -0.531384

9 -1.339975 -0.395147 0.109554

9 -2.993106 -2.476394 0.661314

9 -5.684902 -2.139376 0.567291

9 -6.725846 0.251073 -0.071069

**A**

6 2.212460 12.200687 6.610227

6 -1.318104 8.458697 3.437752

6 1.800396 10.850418 6.682731

6 1.791149 14.141928 5.095861

6 -1.572767 6.094610 3.049698

1 -1.186425 5.078715 3.118498

6 0.922864 10.625506 5.558621

6 3.231782 15.961200 4.408087

1 4.229419 16.319898 4.183070

6 0.684221 14.991475 4.995580

1 -0.302356 14.612879 5.244775

6 0.839866 11.851095 4.857637

1 3.473071 9.982041 9.495612

6 3.108397 10.595705 8.677701

6 0.846032 16.304606 4.590156

1 -0.002327 16.975635 4.505273

6 2.121820 16.801693 4.297769

6 0.219973 9.516767 5.093690

1 0.256846 8.578657 5.640493

6 3.498214 11.940179 8.595190

1 4.157434 12.351450 9.354110

6 2.258882 10.046276 7.728003

1 1.951110 9.006724 7.797969

6 3.057592 14.634786 4.795069

1 3.915181 13.971960 4.859065

6 3.436719 18.647719 3.613648

1 4.112894 18.615439 4.477480

1 3.256966 19.687770 3.339464

1 3.907967 18.129514 2.768812

6 -0.561062 9.621885 3.941159

6 3.057773 12.761987 7.565919

6 -3.245340 7.470700 2.382292

1 -4.225964 7.573459 1.919830

6 -0.817391 7.155068 3.535031

1 0.164615 6.970898 3.960030

6 0.077080 11.972959 3.696657

1 0.037037 12.906672 3.145444

6 -0.615907 10.855596 3.259262

1 -1.195213 10.922896 2.343111

6 -2.575459 8.599454 2.838772

1 -3.040813 9.576684 2.753167

7 1.623708 12.795886 5.499788

8 2.174403 18.100466 3.920681

7 -2.770679 6.227328 2.476947

**A:**PFIB

6 3.366855 12.214074 5.257424

6 -1.126139 8.476091 3.727606

6 3.216895 10.842070 4.951858

6 1.868461 14.206847 5.419170

6 -1.426119 6.378228 2.578655

1 -1.048777 5.587692 1.932590

6 1.816099 10.632758 4.672305

6 2.258976 16.537734 4.896783

1 2.765015 17.280195 4.290629

6 0.979520 14.590093 6.429368

1 0.500047 13.827408 7.035552

6 1.180110 11.887769 4.825011

1 6.447869 9.903161 5.331600

6 5.570276 10.541845 5.308081

6 0.720017 15.929899 6.659544

1 0.031969 16.244050 7.437527

6 1.361189 16.915226 5.898343

6 1.063269 9.518822 4.310466

1 1.538826 8.546285 4.217558

6 5.698093 11.903796 5.618154

1 6.674103 12.300826 5.881905

6 4.334178 10.004654 4.977325

1 4.235736 8.947768 4.745475

6 2.497903 15.186728 4.656652

1 3.177399 14.889185 3.863618

6 1.665830 19.223196 5.469543

1 2.757249 19.203765 5.583764

1 1.280939 20.159756 5.874582

1 1.414844 19.162774 4.402825

6 -0.312211 9.647336 4.103207

6 4.603596 12.758801 5.599641

1 4.704737 13.810294 5.847767

6 -3.167193 7.195460 3.799514

1 -4.188623 7.056869 4.149122

6 -0.623249 7.463995 2.899697

1 0.377672 7.535233 2.485852

6 -0.189427 12.039522 4.607714

1 -0.669029 13.008549 4.698823

6 -0.915989 10.913738 4.254901

1 -1.978741 11.016810 4.055983

6 -2.442019 8.316082 4.180820

1 -2.889565 9.045817 4.848155

7 2.125132 12.835827 5.176547

8 1.047533 18.194003 6.208165

7 -2.680061 6.233455 3.012928

6 -4.902591 1.259714 0.948529

6 -5.660418 0.149810 0.592424

6 -6.968317 0.036134 1.045509

6 -7.509750 1.030279 1.850097

6 -6.736782 2.132948 2.195953

6 -5.425762 2.265701 1.752875

53 -4.259353 3.951655 2.287931

9 -3.655308 1.327461 0.492260

9 -5.140896 -0.801673 -0.178141

9 -7.699327 -1.020440 0.710284

9 -8.762204 0.922442 2.284533

9 -7.295486 3.060487 2.967995

**REFERENCES**

(1) Salunke, J. K.; Wong, F. L.; Feron, K.; Manzhos, S.; Lo, M. F.; Shinde, D., Patil A., Lee, C. S.; Roy, V. A. L.; Sonar, P., Wadgaonkar, P. P. *J. Mater. Chem. C* **4 (5)**, 1009 (2016).

(2) Bonesi, S. M.; Erra-Balsells, R. *J. Lumin.* **93**, 51 (2001).

(3) M. J. Frisch, G. W. Trucks, H. B. Schlegel, G. E. Scuseria, M. A. Robb, J. R. Cheeseman, G. Scalmani, V. Barone, G. A. Petersson, H. Nakatsuji, X. Li, M. Caricato, A. V. Marenich, J. Bloino, B. G. Janesko, R. Gomperts, B. Mennucci, H. P. Hratchian, J. V. Ortiz, A. F. Izmaylov, J. L. Sonnenberg, D. Williams-Young, F. Ding, F. Lipparini, F. Egidi, J. Goings, B. Peng, A. Petrone, T. Henderson, D. Ranasinghe, V. G. Zakrzewski, J. Gao, N. Rega, G. Zheng, W. Liang, M. Hada, M. Ehara, K. Toyota, R. Fukuda, J. Hasegawa, M. Ishida, T. Nakajima, Y. Honda, O. Kitao, H. Nakai, T. Vreven, K. Throssell, J. A. Montgomery, Jr., J. E. Peralta, F. Ogliaro, M. J. Bearpark, J. J. Heyd, E. N. Brothers, K. N. Kudin, V. N. Staroverov, T. A. Keith, R. Kobayashi, J. Normand, K. Raghavachari, A. P. Rendell, J. C. Burant, S. S. Iyengar, J. Tomasi, M. Cossi, J. M. Millam, M. Klene, C. Adamo, R. Cammi, J. W. Ochterski, R. L. Martin, K. Morokuma, O. Farkas, J. B. Foresman, and D. J. Fox, Gaussian 16, revision A.03; Gaussian, Inc.; Wallingford CT, 2016.

(4) R. G. Parr, W. Yang *Density Functional Theory of Atoms and Molecules*. Oxford University Press: New York, 1989.

(5) Casida, M. E. In *Time-Dependent Density-Functional Response Theory for Molecules*; Chong, D. P., Ed.; World Scientific: Singapore, 1995; Vol. 1, p 155-192.

(6) R. Ditchfield, W. J. Hehre, J. A. Pople, *J. Chem. Phys.* **1971**, *54*, 724.

(7) W. J. Hehre, R. Ditchfield, J. A. Pople, *J. Chem. Phys.* **1972**, *56,* 2257.

(8) P. C. Hariharan, J. A. Pople, *Mol. Phys.* **1974**, *27,* 209.

(9) M. S. Gordon, *Chem. Phys. Lett.* **1980**, *76,* 163.

(10) P. C. Hariharan, J. A. Pople, *Theoret. Chim. Acta* **1973**, *28*, 213.

(11) T. H. Dunning Jr., P. J. Hay, *Modern Theoretical Chemistry*, Ed. H. F. Schaefer, III (Plenum, New York, **1976**), vol. 3, p. 1.

(12) P. J. Hay, W. R. Wadt, *J. Chem. Phys.***1985**, *82*, 270.

(13) W. R. Wadt, P. J. Hay, *J. Chem. Phys.***1985**, *82*, 284.

(14) P. J. Hay, W. R. Wadt, *J. Chem. Phys.***1985**, *82*, 2299.

(15) W. J. Hehre, L. Radom, P. v. R. Schleye, J. Pople *Ab initio molecular orbital theory*. John Wiley & Sons: New York, 1986.

(16) J. P. Perdew *Phys. Rev. B.* **1986**, *33*, 8822.

(17) J. P. Perdew, K. Burke, M. Ernzerhof, *Phys. Rev. Lett.* **1997**, *78*, 1396.

(18) S. F. Boys, F. Bernardi, *Mol. Phys.* **1970**, *19*, 553.

(19) M. T. Cancès, B. Mennucci, J. Tomasi, *J. Chem. Phys.***1997**, *107*, 3032.

(20) M. Cossi, V. Barone, B. Mennucci, J. Tomasi, *Chem. Phys. Lett.***1998**, *286*, 253.

(21) B. Mennucci, J. Tomasi, *J. Chem. Phys.***1997**, *106*, 5151

(22) J. E. Carpenter, PhD thesis, University of Wisconsin Madison WI, 1987.

(23) J. E. Carpenter, F. Weinhold, *Theochem-J. Mol. Struct.* **1988**, *46*, 41.

(24) J. P. Foster, F. Weinhold, Natural hybrid orbitals. *J. Am. Chem. Soc.* **1980**, *102*, 7211.

(25) A. E. Reed, F. Weinhold, *J. Chem. Phys.* **1983**, *78*, 4066.

(26) A. E. Reed, F. Weinhold, *J. Chem. Phys.* **1985**, *83*, 1736.

(27) A. E. Reed, R. B. Weinstock, F. Weinhold, *J. Chem. Phys.* **1985**, *83*, 735.

(28) A. E. Reed, L. A. Curtiss, F. Weinhold, *Chem. Rev.* **1988**, *88*, 899.

(29) F. Weinhold, J. E. Carpenter, *The Structure of Small Molecules and Ions*. Plenum, 1988.
